# Tailored PVDF Graft Copolymers via ATRP as High-Performance NCM811 Cathode Binders

**DOI:** 10.1021/acsmaterialslett.3c00485

**Published:** 2023-08-25

**Authors:** Tong Liu, Rohan Parekh, Piotr Mocny, Brian P. Bloom, Yuqi Zhao, So Young An, Bonian Pan, Rongguan Yin, David H. Waldeck, Jay F. Whitacre, Krzysztof Matyjaszewski

**Affiliations:** † Department of Chemistry, Carnegie Mellon University, 4400 Fifth Ave, Pittsburgh, Pennsylvania 15213, United States; ‡ Department of Materials Science and Engineering, Carnegie Mellon University, 5000 Forbes Ave, Pittsburgh, Pennsylvania 15213, United States; § Department of Chemistry, University of Pittsburgh, 219 Parkman Avenue, Pittsburgh, Pennsylvania 15260, United States; ∥ Scott Institute for Energy Innovation, Carnegie Mellon University, 5000 Forbes Ave, Pittsburgh, Pennsylvania 15213, United States

## Abstract

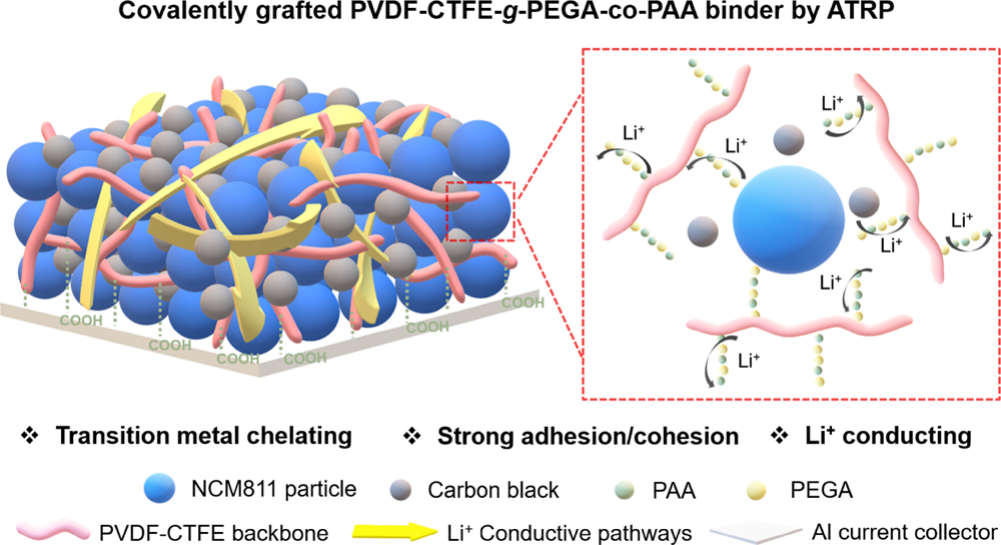

High-nickel layered oxides, e.g., LiNi_0.8_Co_0.1_Mn_0.1_O_2_ (NCM811), are promising candidates for cathode materials in high-energy-density lithium-ion batteries (LIBs). Complementing the notable developments of modification of active materials, this study focused on the polymer binder materials, and a new synthetic route was developed to engineer PVDF binders by covalently grafting copolymers from poly(vinylidene fluoride-*co*-chlorotrifluoroethylene) (PVDF-CTFE) with multiple functionalities using atom transfer radical polymerization (ATRP). The grafted random copolymer binder provided excellent flexibility (319% elongation), adhesion strength (50 times higher than PVDF), transition metal chelation capability, and efficient ionic conductivity pathways. The NCM811 half-cells using the designed binders exhibited a remarkable rate capability of 143.4 mA h g^–1^ at 4C and cycling stability with 70.1% capacity retention after 230 cycles at 0.5 C, which is much higher than the 52.3% capacity retention of nonmodified PVDF. The well-retained structure of NCM811 with the designed binder was systematically studied and confirmed by post-mortem analysis.

Nickel-rich Li(Ni_*x*_Co_*y*_Mn_1–*x*–*y*_O_2_) (*x* ≥ 0.6) (NCM) cathodes are regarded as the predominant cathode materials to meet the ever-increasing demand of high energy density next-generation Li-ion batteries, particularly in the development of electric vehicles.^1−8^ However, undesired structural changes and thermal instability are often observed with increased Ni content, e.g. Ni_0.8_Co_0.1_Mn_0.1_ (NCM811), and can be attributed to transition metal (TM) ion dissolution,^9,10^ phase changes,^11^ gas release,^12^ and microcracks formed on the secondary particles during cycling.^13−15^ As a result, the rapid capacity loss, poor capacity retention, as well as thermal decomposition-related safety issues have hindered the successful practical application of NCM811 cathodes.^16−18^ Several effective strategies have since been proposed to resolve the issues involved with NCM811 cathodes, including metal doping,^19^ surface coating and treatments,^20−22^ gradient structures,^23^ modification of liquid electrolytes,^24−28^ and alternative design of solid electrolytes.^29−31^ An easy and universal remedy is to stabilize the NCM811 cathodes with functional polymer binders. Poly(vinylidene fluoride) (PVDF), because of its remarkable electrochemical stability, has been used in lithium-ion batteries for decades and often serves as a benchmark material. However, the lack of functional groups, poor adhesion, poor conductivity, and limited interactions with active particles make it no longer the optimal choice for more challenging cathodes.^32−34^ Thus, identifying best polymer binders is paramount to increasing performance metrics and ensuring the stability of the cathodes, particularly, to achieve higher energy density with a higher content of active material and lower content of the polymer binder.^35−37^

Ideal binders must feature (i) high chemical and electrochemical stability, (ii) good mechanical properties, e.g., high modulus, stretchability and flexibility, strong adhesion, and cohesion, and (iii) high electronic and/or ionic conductivities. Other desired properties may include oxygen scavenging, transition metal chelation, or HF neutralization properties.^38−44^ Several tailored polymer binders for improving performance of NCM811 cathodes have been designed and used in the past.^45−47^ For example, a modulated 3D network binder with a stiff polyimide backbone and flexible siloxane segments was reported, offering flexibility and mechanical properties to ensure cathode-electrolyte interface stability of NCM811 cathodes.^48^ Synergistic structural effects can be achieved with amphiphilic copolymers, *e.g*. bottlebrush polymers comprising hydrophobic polynorbornene and poly(acrylic acid) side chains. The nonswelling hydrophobic backbone provided structural integrity, while poly(acrylic acid) side chains improved the binding strength and enabled high active content and high mass loading of NCM811 cathodes.^49^ In the context of PVDF modification, vinylphenol-grafted PVDF binders showed improved NCM cathode stability arising from a vinylphenol-mediated decrease in O_2_ generation, effectively suppressing oxygen release in the NCM cathode.^50^ Despite the marked improvement in the NCM cathode, these binders require multistep tedious synthesis and/or involve poorly controlled polymerization with broad molecular weight distribution, nonuniform chain length, random branching, *etc*. Moreover, some studies reported use of a physical mixture of binders by simply blending several polymers, which can result in poor uniformity and coverage, phase separation/aggregation, and overall heterogeneity of the cathode materials.^51,52^

Herein, we present a robust and feasible approach for re-engineering PVDF binders by covalently grafting tunable functional polymers under a controlled polymerization process. This preserves the original stabilities and properties of the PVDF binders as well as provides new and desirable interactions with the active particles. The previously demonstrated modification of PVDF often required harsh conditions, such as high energy radiation, high catalyst loadings, and high temperatures over a long time to activate C–F bond cleavage.^53−55^ Also, base-promoted dehydrofluorination of PVDF may drastically reduce its solubility and processability.^50^ There is a clear need for simplified synthetic procedures, which are important for screening a range of binders and ultimate optimization. In this work, poly(vinylidene fluoride-*co*-chlorotrifluoroethylene) (PVDF-CTFE), a PVDF derivative, was employed. The incorporated CTFE (10 wt %) provides C–Cl sites with lower bond dissociation energy than C–F in PVDF, which facilitates the initiation of grafted polymer chains.^56^ Atom transfer radical polymerization (ATRP), the controlled radical polymerization technique, was used to obtain well-controlled grafted chains with low dispersity and desired length and architecture.^57−63^ We used a light-mediated ATRP with Eosin Y as a photocatalyst for its excellent oxygen-tolerance, temporal control, fast polymerization, as well as greatly reduced copper catalyst loadings.^64^ A random copolymer of oligo(ethylene glycol) methyl ether acrylate and *tert*-butyl acrylate (PEGA-*co*-P*t*BA) was first grafted from the PVDF-CTFE backbone; then, after hydrolysis of P*t*BA to poly(acrylic acid) (PAA), the targeted PVDF-CTFE-*g*-PEGA-*co*-PAA graft copolymers were obtained (Figure 1a). This design offers the following advantages: (i) the covalently linked PEGA-*co*-PAA grafts are inseparable from the PVDF backbone, which prevents macrophase separation/aggregation. It is clearly superior to physically blended polymer systems in providing homogeneous coverage and overall uniform distribution of the cathode materials; (ii) the poly(ethylene oxide) conductive segments in PEGA should significantly improve the Li-ion diffusion and transport (Figure 1b), enabling better rate capabilities; (iii) the chelation of −COOH groups from PAA side chains to transition metal (TM) ions effectively mitigates TM dissolution, therefore preventing phase transition of NCM811; (iv) hydrogen bonding of −COOH groups affords improved adhesion, cohesion, and stretchability of the polymer binders which should maintain the overall mechanical property and stability of the cathodes (Figure 1b).

**Figure 1:**
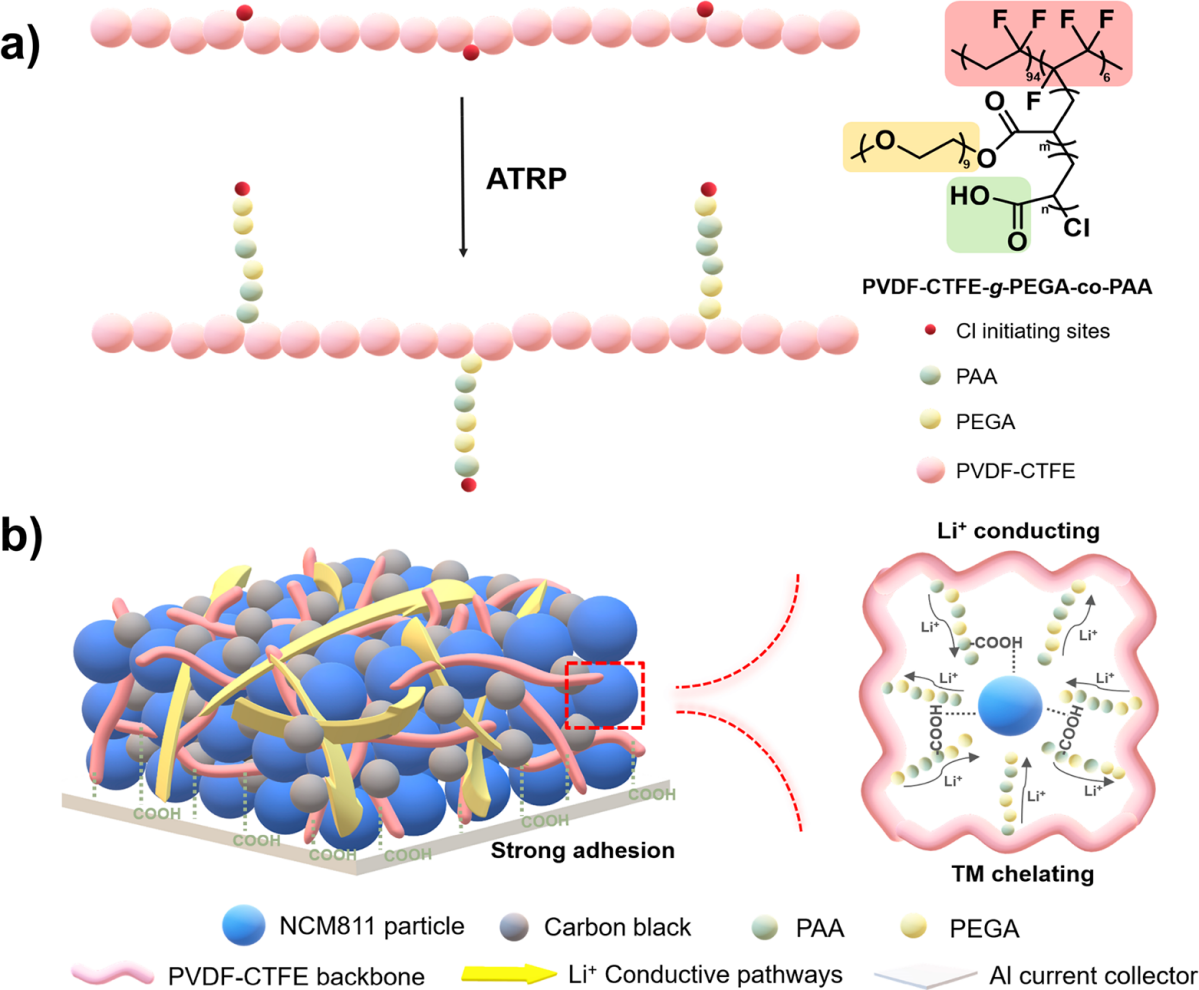
a) Comb-like structure of functionalized PVDF via “grafting from” method by ATRP. b) Scheme of NCM811 cathodes with the PVDF-CTFE-*g*-PEGA-*co*-PAA binder with ion-conductive pathways and transition metal chelation sites.

**Synthesis and physicochemical properties of the grafted PVDF-CTFE binder**. The grafting from PVDF-CTFE was performed under green light irradiation (520 nm, 9.0 mW cm^–2^) via ATRP using PVDF-CTFE as the macroinitiator, Eosin Y as the photocatalyst, and CuCl_2_/Me_6_TREN (Me_6_TREN = tris[2-(dimethylamino)ethyl]amine) as the dual catalytic system, enabling a relatively oxygen-tolerant polymerization condition with short 5 min nitrogen-purging (Figure 2a).^64,65^ By tuning the ratio between C–Cl initiating sites in PVDF-CTFE and the monomer feed ratio, we effectively designed and controlled the side chain length, resulting in a tailorable weight percent of the grafted chains. Three types of grafted polymers were made to study the effect of the functional groups, including the homopolymer of P*t*BA, PEGA, and a random copolymer PEGA-*co*-P*t*BA. The resulting PVDF-CTFE-*g*-P*t*BA and PVDF-CTFE-*g*-PEGA*-co-Pt*BA were then exposed to trifluoroacetic acid (TFA) to obtain PAA segments that acted as binding groups to the electrodes, while PEGA contributed to the ion-transport pathways. Note, the weight percent of the grafted polymers was varied with 20, 70, and 89 wt % grafted chains of the final polymer binder, and their performances were assessed.

**Figure 2:**
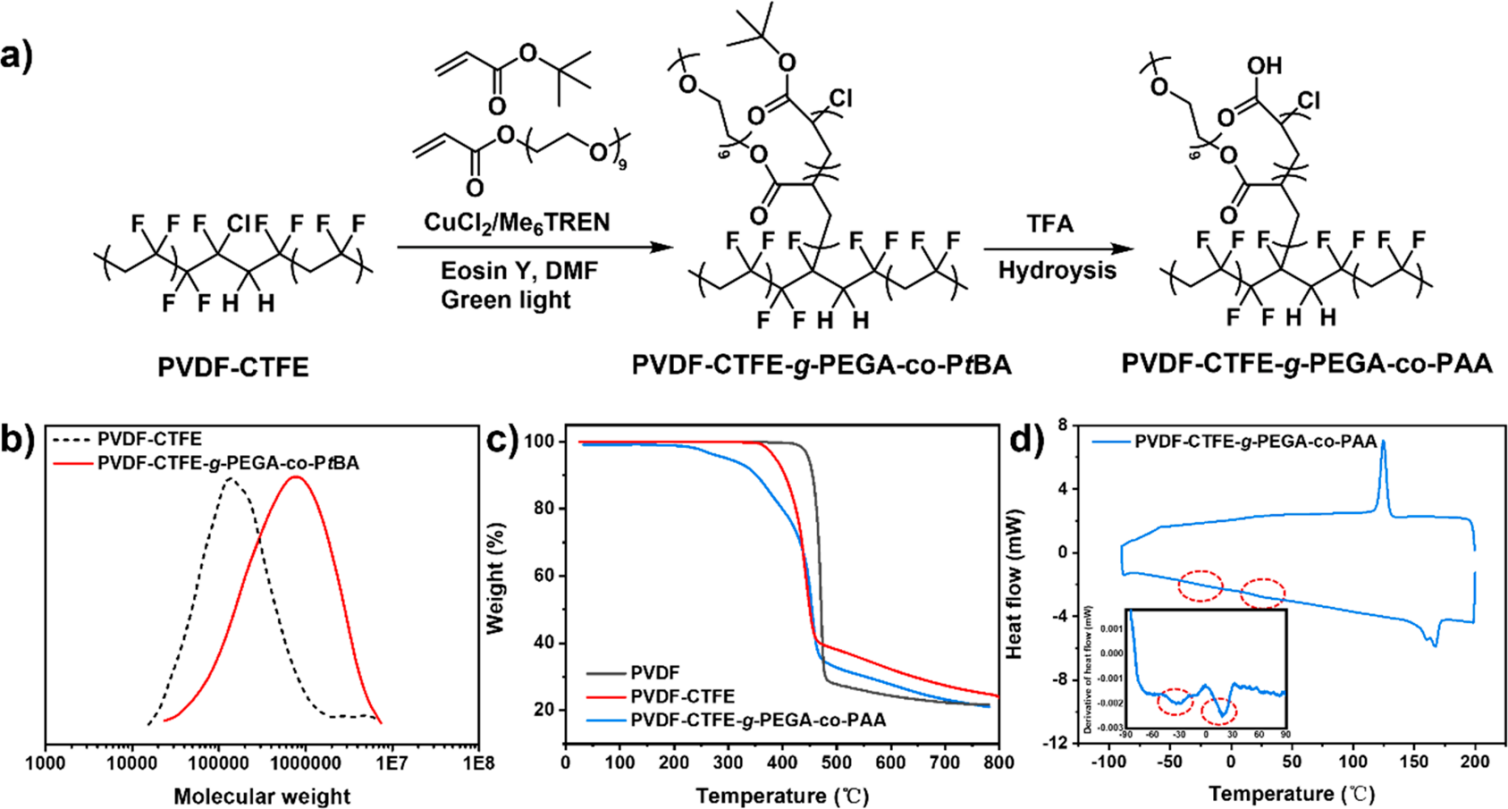
a) Synthetic route for grafting PEGA-*co*-PAA random copolymers from PVDF-CTFE backbones via ATRP under fast oxygen-tolerant conditions. b) Gel permeation chromatography (GPC) traces of PVDF-CTFE before (black) and after (red) the grafting. c) Thermogravimetric analysis (TGA) curves of PVDF (black), PVDF-CTFE (red), and PVDF-CTFE-*g*-PEGA-*co*-PAA (blue). d) Differential scanning calorimetry (DSC) and derivative DSC (inset) curves of PVDF-CTFE-*g*-PEGA-*co*-PAA.

After purification of the synthesized polymers, NCM811 cathodes were prepared with a ratio of the active material/carbon black/polymer binder in a 85/10/5 wt % ratio with the different polymer binders. The NCM811 cathodes were then tested in Li|NCM811 half cells by comparing the rate performance to select the optimal composition of the polymer binders (Table S1). PVDF and PVDF-CTFE were used as important controls to compare the performance of the newly functionalized binders. Rate capability results showed that both PVDF-CTFE-*g*-PEGA (20 wt % grafts) and especially PVDF-CTFE-*g*-PEGA-*co*-PAA (20 wt % grafts) delivered higher discharge capacities than PVDF and PVDF-CTFE. Conversely, PVDF-CTFE-*g*-PAA binders resulted in much lower discharge capacities, in both the 20 and 70 wt % grafts, which was attributed to the large amount of strongly binding PAA segments leading to sluggish Li^+^ diffusion.^66^ PVDF-CTFE-*g*-PEGA (89 wt % grafts) resulted in severe delamination of the prepared NCM811 cathodes due to relatively poor mechanical properties and the absence of strong binding affinity of PEGA. In contrast, mechanical properties of PVDF-CTFE-*g*-PEGA-*co*-PAA (20 wt % grafts) were dominated by the robust PVDF-CTFE-based backbone. Therefore, it was selected as the optimal composition for material characterization and further electrochemical testing in a subsequent study. The proton nuclear magnetic resonance (^1^H NMR) spectra of the graft copolymers are shown in Figure S1.

Gel permeation chromatography (GPC) measurements showed a clear shift to higher molecular weight from PVDF-CTFE (black) to PVDF-CTFE-*g*-PEGA-*co*-PAA (red), indicating the successful grafting of the copolymer (Figure 2b). Fourier-transform infrared spectroscopy (FTIR) of PVDF, PVDF-CTFE, and PVDF-CTFE-*g*-PEGA-*co*-PAA further confirmed the functionalization of PVDF-CTFE with −COOH, C=O, and C–O functional groups originating from the grafted PEGA-*co*-PAA chains (Figure S2). In Figure 2c, thermogravimetric analysis (TGA) showed a corresponding weight loss of ∼20 wt %, in accordance with the graft copolymer content calculated from conversion of the polymerization. Although the onset decomposition temperature of PVDF-CTFE-*g*-PEGA-*co*-PAA (250 °C) was lower than those of PVDF (450 °C) and PVDF-CTFE (370 °C), this behavior could arise from intrinsic thermal instability of PAA and PEGA. To further study and compare the thermal behavior of the three copolymers, differential scanning calorimetry (DSC) was conducted (Figure 2d and Figure S3). The melting temperatures of PVDF, PVDF-CTFE, and PVDF-CTFE-*g*-PEGA-*co*-PAA were 170, 166, and 167 °C, respectively. The glass transition temperatures (*T*_g_) of PVDF (−41 °C) and PVDF-CTFE (−33 °C) were similar, whereas in PVDF-CTFE-*g*-PEGA-*co*-PAA, two *T*_g_ were observed, one at −33 °C corresponding to the PVDF-CTFE backbone and the other at 18 °C was due to the grafted random copolymer of PEGA-*co*-PAA.

**Mechanical property and adhesion**. Determination of mechanical properties of polymeric binders is critical for evaluating their performance because the binding strength between the particles, as well as against the current collector, is crucial for ensuring the structural integrity of the cathodes. Moreover, the flexibility and elasticity of the binders are important to accommodate volume changes during cycling and possible mechanical shear/bending of the electrodes. To examine the adhesion strength of the NCM811 cathodes prepared with PVDF (black), PVDF-CTFE (red), and PVDF-CTFE-*g*-PEGA-*co*-PAA (blue) binders, a 180° peel test was performed to peel the NCM811 cathodes from the aluminum (Al) current collector using 3 M Scotch 600 tape (Figure 3a and Figure S4). Under the testing conditions, PVDF and PVDF-CTFE showed nearly no adhesion with a peel strength of 0.021 and 0.025 N/cm, respectively. However, PVDF-CTFE-*g*-PEGA-*co*-PAA exhibited a 50 times larger peel strength of 1.1 N/cm. The increased adhesion strength is attributed to the −COOH groups in grafted PAA. Figure 3b displays strain–stress curves of bulk polymer binder films, which reveal a significant difference in deformation behavior for PVDF, PVDF-CTFE, and PVDF-CTFE-*g*-PEGA-*co*-PAA. The PVDF binder broke at a yield point of 17.6%, indicating a hard and brittle plastic property. Conversely, PVDF-CTFE showed necking after the yield point and strain hardening under uniaxial tension with a break at 92.1%. The deformation of PVDF-CTFE-*g*-PEGA-*co*-PAA, however, displayed superior strain, exceeding 319%. The larger deformation ratio was attributed to the grafted PEGA-*co*-PAA, generating a more elastomer-like plastic material with remarkably larger toughness (23.69 × 10^6^ J/m^3^) compared to PVDF (2.23 × 10^6^ J/m^3^) and PVDF-CTFE (10.04 × 10^6^ J/m^3^) (Figure S5). Scanning electron microscopy (SEM) images of the NCM811 cathodes before cycling are shown in Figure 3c-e and were used to compare the surface morphology. With the PVDF binder, the NCM811 cathode showed a compact and flat surface; however, a relatively nonuniform particle distribution and particle aggregation were observed (Figure 3c and Figure S6a). In contrast, the NCM811 cathode with PVDF-CTFE and PVDF-CTFE-*g*-PEGA-*co*-PAA binders exhibited a more homogeneous dispersion and less agglomeration of the particles (Figure 3d and e; Figure S6b-c). The cross-sectional SEM of the NCM811 cathodes before cycling with the three binders exhibited rather similar morphology, where randomly distributed nanopores were observed from the cross-section (Figure S6d-f). Micro Computed Tomography (Micro-CT) was performed for the NCM811 electrodes prepared with the three binders, showing that there were no significant variations of electrode porosities by PVDF (36.4%), PVDF-CTFE (38%), and PVDF-CTFE-*g*-PEGA-*co*-PAA (34.6%) binders (Figures S7–S9).

**Figure 3:**
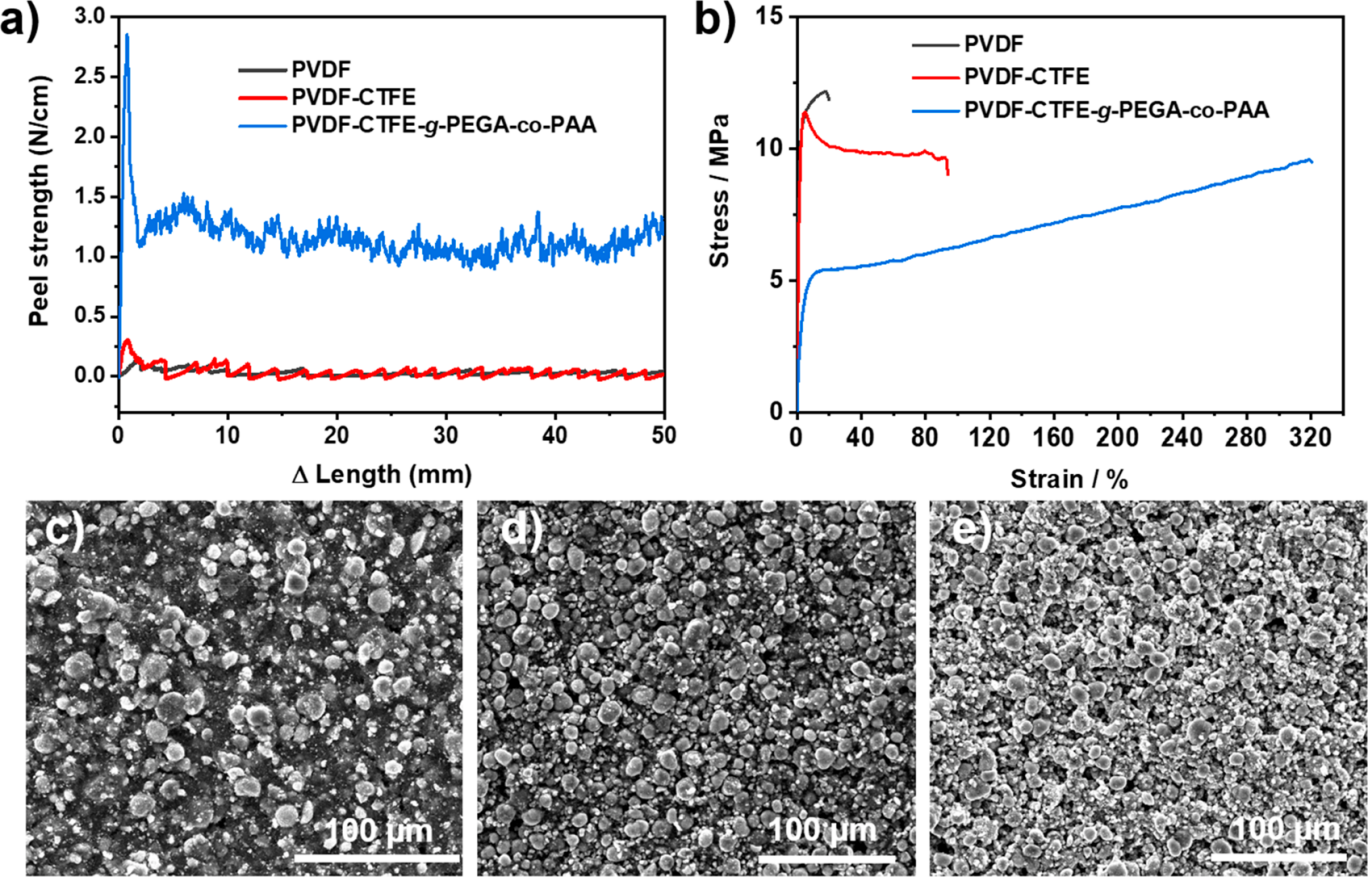
a) Peel strength of NCM811 cathodes on the Al current collector with PVDF (black), PVDF-CTFE (red), and PVDF-CTFE-*g*-PEGA-*co*-PAA (blue) binders. b) Stress–strain curves of PVDF (black), PVDF-CTFE (red), and PVDF-CTFE-*g*-PEGA-*co*-PAA (blue). Top view SEM images of NCM811 cathodes before cycling with c) PVDF, d) PVDF-CTFE, and e) PVDF-CTFE-*g*-PEGA-*co*-PAA binders.

**Electrochemical performance of Li|NCM811 cell**s. To evaluate the efficacy of the PVDF-CTFE-*g*-PEGA-*co*-PAA binder, a NCM811/carbon black/polymer binder ratio (93/4/3 wt %) was used. To form a more active material, Li|NCM811 half cells were assembled using 1 M LiPF_6_ in ethylene carbonate/ethyl methyl carbonate (EC/EMC = 3/7) as the liquid electrolyte. Figure 4a shows the rate performance of the Li|NCM811 cells at 0.1C, 0.2C, 0.5C, 1C, 2C, and 4C. While the discharge capacities of the NCM811 cathodes with PVDF (black) and PVDF-CTFE (red) binders were comparable, the PVDF-CTFE-*g*-PEGA-*co*-PAA binder (blue) showed much higher discharge capacities, particularly at 1C (171.7 mAh/g), 2C (161.9 mAh/g), and 4C (143.4 mAh/g). The excellent rate capability suggested faster Li ion transport associated with the grafted PEGA-*co*-PAA copolymers. Figure 4d-f) shows the corresponding voltage-capacity curve from the rate test and further demonstrates improved mass transport and less polarization of the NCM811 cathode using the PVDF-CTFE-*g*-PEGA-*co*-PAA binder. A similar trend was also observed in experiments using different weight contents of the active material (Figure S10). It is important to note that a physical mixture of PVDF-CTFE/PEGA/PAA was also prepared and compared, using the same molecular weight and ratio of the three components, as in the grafted PVDF-CTFE-*g*-PEGA-*co*-PAA. The rate performance of the covalently grafted binder showed higher capacities than the physical mixture binder, especially at higher C rates: 1C, 2C, and 4C. This confirmed the advantage and significance of the covalent grafting method by ATRP for PVDF re-engineering (Figure S11 and Figure S12).

**Figure 4:**
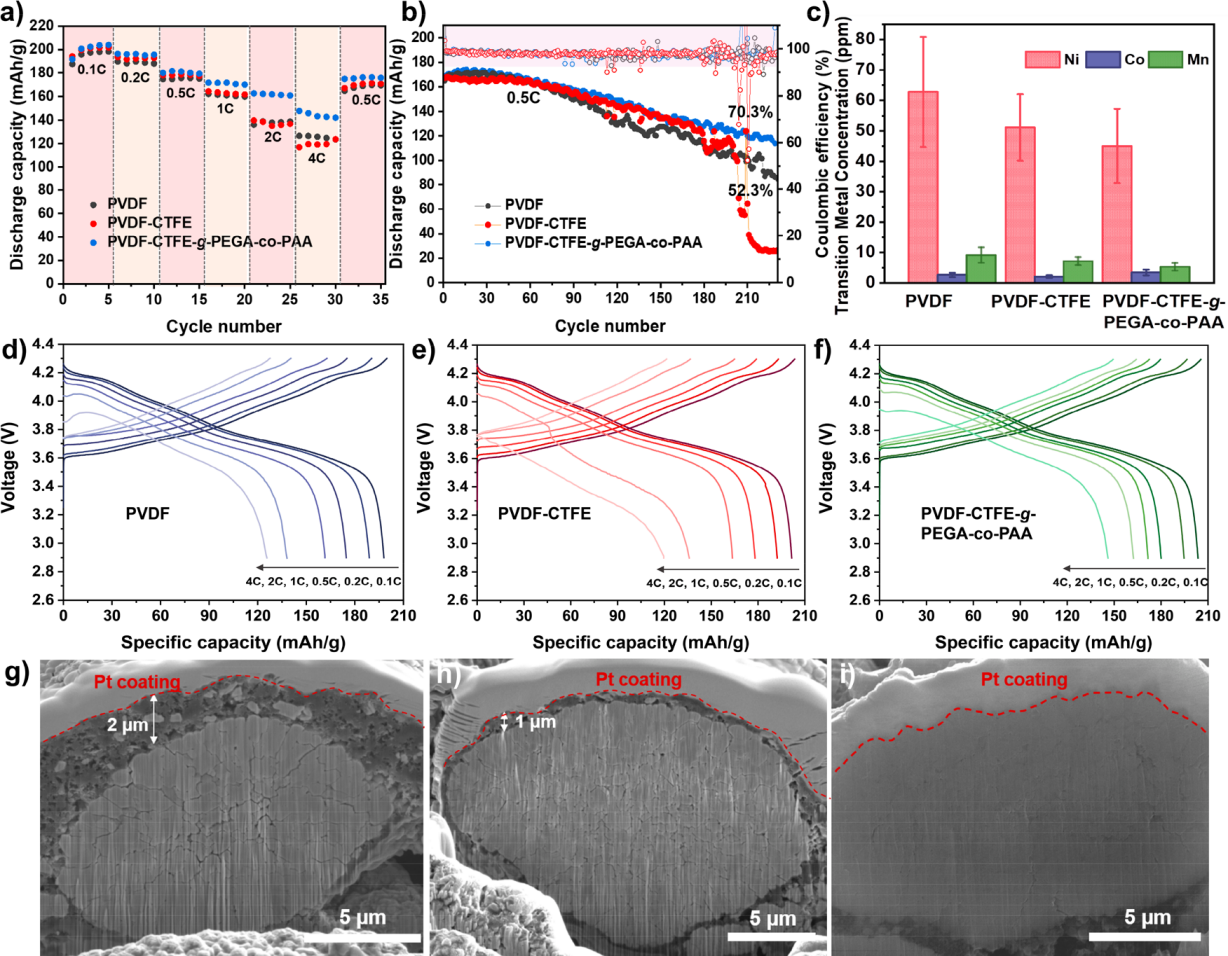
a) Rate performance of Li|NCM811 cells with PVDF (black), PVDF-CTFE (red), and PVDF-CTFE-*g*-PEGA-*co*-PAA (blue) binders at 0.1C, 0.2C, 0.5C, 1C, 2C, and 4C in the voltage range of 2.9–4.3 V. b) Discharge capacity and cycle life of Li|NCM811 cells using PVDF (black), PVDF-CTFE (red), and PVDF-CTFE-*g*-PEGA-*co*-PAA (blue) binders at 0.5C. c) ICP-MS analysis of transition metal content from the disassembled Li|NCM811 cells after cycling. Corresponding voltage-capacity profiles of rate testing of Li|NCM811 cells using d) PVDF, e) PVDF-CTFE, and f) PVDF-CTFE-*g*-PEGA-*co*-PAA binders. FIB-SEM cross-section images of NCM811 cathodes after 250 cycles using g) PVDF, h) PVDF-CTFE, and i) PVDF-CTFE-*g*-PEGA-*co*-PAA binders.

The long-term cycling stabilities of the Li|NCM811 cells with PVDF, PVDF-CTFE, and PVDF-CTFE-*g*-PEGA-*co*-PAA binders were tested at 0.5C (Figure 4b). After 230 cycles, the PVDF-CTFE-*g*-PEGA-*co*-PAA cathodes (blue) showed the highest capacity retention of 70.3%, exhibiting a stable cycling performance with negligible fluctuations, whereas PVDF cathodes (black) presented severe fluctuation in discharge capacities after 100 cycles, followed by only 52.3% capacity retention after 230 cycles. The PVDF-CTFE cathodes (red) showed comparable cycling stability to that of PVDF-CTFE-*g*-PEGA-*co*-PAA until 172 cycles; however, the capacities and Coulombic efficiency exhibited severe variation with a drastic decrease; the capacities dropped to ∼30 mAh/g after 190 cycles. A similar trend was observed for the three binders in the long term cycling of Li|NCM811 cells at 1C with a higher areal capacity of ∼1.1 mAh/cm^2^ (Figure S13). The discharge/charge profile evolution at different cycle numbers is presented in Figure S14. It showed that not only the NCM811 cathodes with the PVDF-CTFE-*g*-PEGA-*co*-PAA binder possessed higher reversible specific capacities than PVDF-CTFE and PVDF but also the initial overpotential for the delithiation at longer cycling (200 cycles) was significantly higher, suggesting improved kinetics of NCM811 cathodes with the PVDF-CTFE-*g*-PEGA-*co*-PAA binder. The Nyquist plots and fitting results of electrochemical impedance spectra (EIS) are shown in Figures S15, S16 before and after long-term cycling. As illustrated, the charge transfer resistance (*R*_ct_) of PVDF-CTFE-*g*-PEGA-*co*-PAA (74.35 Ω) was smaller than that of PVDF (77.05 Ω) and PVDF-CTFE (113.5 Ω) before cycling, indicating faster Li^+^ diffusion in the grafted PEGA conductive segments. The Z′ vs ω ^–1/2^ plots at low frequency suggested the solid ionic diffusion ability of PVDF-CTFE-*g*-PEGA-*co*-PAA (Figure S17), which resulted in the highest apparent lithium diffusion coefficient *D*_app_ (1.03 × 10^–14^ cm^2^ s^–1^) compared to PVDF (7.84 × 10^–15^ cm^2^ s^–1^) and PVDF-CTFE (3.04 × 10^–15^ cm^2^ s^–1^). Furthermore, a small *R*_f_ (110.0 Ω) after 250 cycles revealed better cathode-electrolyte interface stability using the PVDF-CTFE-*g*-PEGA-*co*-PAA binder.

**Post-mortem analysis of understanding the cycled NCM811 cathodes**. To understand the possible degradation mechanism of NCM811 cathodes, post-mortem analysis was conducted. SEM images of the NCM811 surface morphology postcycling are shown in Figure S18. Significant cracking was observed for cathodes made with the PVDF and PVDF-CTFE binders; however, the PVDF-CTFE-*g*-PEGA-*co*-PAA binder showed a remarkably similar surface morphology to that before cycling without cracks. To assess the internal stresses and crack formation of the cycled NCM811 particles, Focused Ion Beam Scanning Electron Microscopy (FIB-SEM) was conducted to examine the cross-section of cycled NCM811 particles (Figure 4g-i). It is clearly seen that NCM811 cathodes with the PVDF binder developed intergranular cracks and enlarged pores from the center of the particle, whereas the PVDF-CTFE binder had much fewer microcracks. In sharp contrast, NCM811 cathodes with the PVDF-CTFE-*g*-PEGA-*co*-PAA binder showed a more dense and integrated cross-section morphology with only minor pore enlargement compared to that before cycling, demonstrating alleviated lattice expansion and contraction of the NCM811 particle grains formed through repeated cycling.^67^ Surprisingly, the formation of an interface layer was observed with a thickness of 2 and 1 μm for PVDF and PVDF-CTFE prepared NCM811 cathodes, respectively. Such thick cathode electrolyte interphase (CEI) contrasted with the common belief that the CEI layers have a general thickness of a few nanometers.^68^ It is very likely induced by the noncryo environment and exposure to air/moisture during sample transfer and handling for FIB-SEM analysis.^69^ Nonhomogeneous conductive carbon and binder coverages could also contribute to the thickness of the observed interface layer. Nevertheless, the PVDF-CTFE-*g*-PEGA-*co*-PAA binder showed negligible buildup of such an interface, indicating less efficient CEI generation over cycling and a more uniform carbon/binder coverage. Other parts of the disassembled cells, e.g., Li metal and separator, were collected for inductively coupled plasma mass spectrometry (ICP-MS) analysis; see the Supporting Information for more details regarding this process. Figure 4c shows the concentrations of Ni, Co, and Mn on the Li metal and separator. The Ni and Mn concentrations follow the trend of PVDF > PVDF-CTFE > PVDF-CTFE-*g*-PEGA-*co*-PAA, indicating improved metal-chelating properties of the PVDF-CTFE-*g*-PEGA-*co*-PAA binder owing to the grafted PAA chains. Although the Co concentration was slightly higher for the PVDF-CTFE-*g*-PEGA-*co*-PAA than PVDF and PVDF-CTFE samples, the correlation of Co dissolution to the structural stability of NCM811 cathodes and Li|NCM811 cells was less significant compared to Mn and Ni.^70,71^

To further analyze the structure of the NCM811 cathodes, powder X-ray diffraction (XRD) was performed (Figure S19) before and after long-term cycling (250 cycles). The initial and cycled cathodes with PVDF, PVDF-CTFE, and PVDF-CTFE-*g*-PEGA-*co*-PAA binders showed X-ray diffraction patterns which match a hexagonal *α*-NaFeO_2_ structure without any impurity peaks, with the exception of an Al peak associated with the current collector.^72^ Following cycling, a pronounced shift to lower 2θ for the 003 peak for PVDF (Δ2θ = 0.05), PVDF-CTFE (Δ2θ = 0.16), and PVDF-CTFE-*g*-PEGA-*co*-PAA (Δ2θ = 0.01) was observed. The 003 lattice plane corresponds to the *c*-axis of the NCM811, and the corresponding shift to lower 2θ represents a volume increase in the structure, as well as a decrease in the Li-ion intercalation.^73^ The smaller 2θ shift for PVDF-CTFE-*g*-PEGA-*co*-PAA thus corroborates the FIB-Image analysis and demonstrates suppression of structural transformations of the NCM811.

To assess the effect of binder composition on NCM811 cathode degradation and surface chemistry, the fresh and cycled NCM811 cathodes were analyzed by X-ray photoelectron spectroscopy (XPS). Figure 5 shows C 1s and F 1s spectra of NCM811 using a, d) PVDF; b, e) PVDF-CTFE; and c, f) PVDF-CTFE-*g*-PEGA-*co*-PAA binders before (top) and after (bottom) cycling. The C 1s spectra were fit to a series of peaks at 284.8, ∼286.4, ∼288.4, and ∼291 eV and correspond to C–C, C–O, C=O, and CF_2_, respectively. Note, the red line is an envelope fitting to the spectra. Upon cycling the relative contributions to the C 1s spectra change, a new peak emerges (∼289.3 eV), which was associated with carbonate species. This behavior is consistent with that shown in other reports^74,75^ and is attributed to the formation of a CEI layer. Indeed, CEI growth on NCMs is a well-accepted degradation mechanism and can lead to large impedance hikes and decreased electrochemical performance.^76,77^Figure 5g shows that the relative peak contributions with the PVDF-CTFE-*g*-PEGA-*co*-PAA binder mostly persist upon cycling, whereas C–O, C=O, and CO_3_ contributions from the various complex organic/inorganic interphase species begin to dominate for NCM811 cathodes with PVDF and PVDF-CTFE binders, implying that the thickness of the CEI layer is minimized for the PVDF-CTFE-*g*-PEGA-*co*-PAA binder upon cycling compared to that with PVDF-CTFE and PVDF. The CEI formation and resulting thickness among the different binders were in good agreement with the FIB-SEM image interpretations shown in Figure 4g-i. Further information about the CEI layer can be inferred from the O 1s and F 1s spectra. Figure S20 shows the spectral profile of the O 1s before and after cycling, fitted to a series of three separate resolvable peaks at ∼529.5, ∼ 532.0, and ∼533.6 eV which are attributed to metal oxide, organic oxygen content, and Li_*x*_PO_*y*_F_*z*_, respectively. Upon cycling, a peak associated with Li_*x*_PO_*y*_F_*z*_ emerges. Figure 5d-f shows F 1s spectra fitted to three peaks at ∼685, ∼ 686.3, and ∼688 eV and are associated with metal-fluoride, Li_*x*_PO_*y*_F_*z*_, and polymeric fluorine, respectively.^74^ Thus, the O 1s and F 1s spectra collectively confirm the presence of Li_*x*_PO_*y*_F_*z*_ upon cycling. The relative atomic percent ratio of polymeric fluorine to Li_*x*_PO_*y*_F_*z*_ and M-F corroborates the C 1s findings that the thickness of the CEI layer for PVDF and PVDF-CTFE binders is greater than that for PVDF-CTFE-*g*-PEGA-*co*-PAA (Figure 5g). Moreover, previous experiments have shown that the interfacial impedance and hence general performance of NCM811 cathodes were susceptible to the amount of Li_*x*_PO_*y*_F_*z*_ in relation to LiF and Li_2_CO_3_ in the CEI layer.^74^ In this report, the relative atomic percent ratio of Li_*x*_PO_*y*_F_*z*_ and MF_2_/LiF to C–F from the polymer binders followed the trend, PVDF > PVDF-CTFE > PVDF-CTFE-*g*-PEGA-*co*-PAA, further confirming that the formation of Li_*x*_PO_*y*_F_*z*_ can lead to deteriorated performance of the NCM811 cathode.

**Figure 5:**
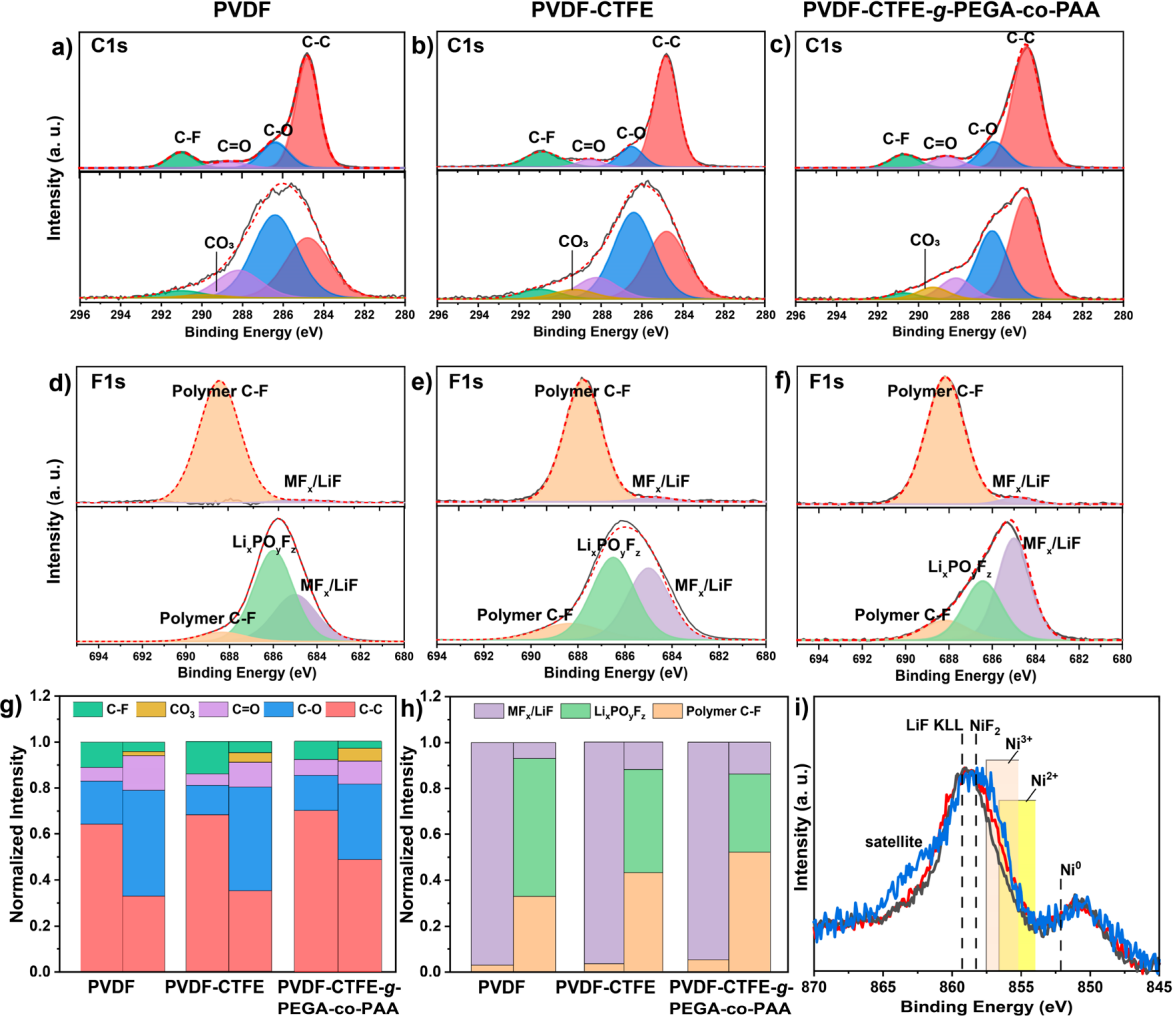
XPS analysis of NCM811 electrodes with PVDF, PVDF-CTFE, and PVDF-CTFE-*g*-PEGA-*co*-PAA binders before and after 250 cycles: (a-c) C 1s spectra and d-f) F 1s spectra; g) Normalized quantitative analysis of the different carbon-containing surface species calculated from C 1s spectra fitting; h) Normalized quantitative analysis of the different fluorine-containing surface species calculated from F 1s spectra fitting; (i) Ni 2p spectra of NCM811 cathodes after 250 cycles with PVDF (black), PVDF-CTFE (red), and PVDF-CTFE-*g*-PEGA-*co*-PAA (blue) binders.

To assess the quality of the cathodes following cycling, intensity normalized Ni 2p_3/2_ spectra for PVDF (black), PVDF-CTFE (red), and PVDF-CTFE-*g*-PEGA-*co*-PAA (blue) binder prepared cathodes were collected and are overlaid in Figure 5i. Because of the large amount and subsequent complex satellite structures of possible Ni compositions, which give rise to significant spectral overlap, multicomponent fitting of the spectra was not performed. Instead, individual regions comprising different compositions are highlighted, in accordance with previous literature precedence.^77,78^ The major differences among the spectra are represented by a shoulder at 856.5 eV, attributed to Ni^3+^ species or NiOH_2_,^79^ and a corresponding satellite feature at 863 eV. The relative intensity ratio of the Ni^3+^ region in the XPS spectra to that of the Ni^2+^ region correlated well with the improved stability of Zr substituted NCM811 over unsubstituted analogs.^72^ Indeed, the presence of Ni^2+^ has been associated with the plausible Li^+^/Ni^2+^ ion mixing and irreversible phase transition of NCM811.^80^ In this work, the Ni^3+^: Ni^2+^ follows the trend PVDF-CTFE-*g*-PEGA-*co*-PAA > PVDF-CTFE > PVDF binders, similar to the improved long-term cycling stability suggested in Li|NCM811 cells. Therefore, the XPS analysis implies that PVDF-CTFE-*g*-PEGA-*co*-PAA showed improved suppression of complex organic/inorganic interfacial species formation, inhibited byproduct generation, and preserved the ordered phase of NCM811 compared to PVDF and PVDF-CTFE.

In summary, we have explored a new synthetic route to re-engineer PVDF binders by grafting from PVDF-CTFE backbones to obtain a comb-like polymer architecture with multiple functionalities. The grafted architecture and controlled polymerization technique provide advantages over physically blended/mixed polymer systems by achieving better uniformity of the polymers, preventing phase separation/aggregation, and thus providing more homogeneous morphology of the cathode materials. The grafted chains were designed to comprise Li-ion conducting PEGA and strongly binding PAA as random copolymer side chains. The conducting PEGA segments contributed to facilitated Li-ion transport and diffusion kinetics, giving rise to excellent rate performance (4C with a discharge capacity of 143.4 mAh/g) with a high active material content (93 wt %) and low binder content (4 wt %), as compared to control measurements made on PVDF and PVDF-CTFE binders. Moreover, the relative softness of PEGA contributed to the stretchability and flexibility of the binder (319% elongation), and the PAA segments provided strong binding and adhesion of the electrode (∼50 times larger peel strength than that of PVDF), as well as enabling transition metal chelation to inhibit phase transitions caused by TM dissolution. The systematic postcycling analysis indicated that the PVDF-CTFE-*g*-PEGA-*co*-PAA binder was beneficial for controlling CEI formation as well as maintaining the stability of NCM811 by suppressing phase transition and degradation.

This study demonstrates the potential of grafting techniques for creating advanced polymer binders with designed functionalities, tunable grafting density, and variable grafts weight ratio, providing new pathways toward high-energy-density batteries.

## Supporting Information Available

Materials, experimental section, and additional data of synthesis and characterization (PDF)

## References

[ref1] Li W.; Erickson E. M.; Manthiram A. High-nickel layered oxide cathodes for lithium-based automotive batteries. Nature Energy 2020, 5, 26–34. 10.1038/s41560-019-0513-0.

[ref2] Lee W.; Muhammad S.; Sergey C.; Lee H.; Yoon J.; Kang Y.-M.; Yoon W.-S. Advances in the Cathode Materials for Lithium Rechargeable Batteries. Angew. Chem., Int. Ed. 2020, 59, 2578–2605. 10.1002/anie.201902359. 31034134

[ref3] Grey C. P.; Hall D. S. Prospects for lithium-ion batteries and beyond—a 2030 vision. Nat. Commun. 2020, 11, 6279 10.1038/s41467-020-19991-4. 33293543 PMC7722877

[ref4] Manthiram A.; Knight J. C.; Myung S.-T.; Oh S.-M.; Sun Y.-K. Nickel-Rich and Lithium-Rich Layered Oxide Cathodes: Progress and Perspectives. Adv. Energy Mater. 2016, 6, 1501010 10.1002/aenm.201501010.

[ref5] Manthiram A. A reflection on lithium-ion battery cathode chemistry. Nat. Commun. 2020, 11, 1550 10.1038/s41467-020-15355-0. 32214093 PMC7096394

[ref6] Julien C. M.; Mauger A. NCA, NCM811, and the Route to Ni-Richer Lithium-Ion Batteries. Energies 2020, 13, 6363 10.3390/en13236363.

[ref7] Assat G.; Tarascon J.-M. Fundamental understanding and practical challenges of anionic redox activity in Li-ion batteries. Nature Energy 2018, 3, 373–386. 10.1038/s41560-018-0097-0.

[ref8] Tarascon J.-M. Material science as a cornerstone driving battery research. Nat. Mater. 2022, 21, 979–982. 10.1038/s41563-022-01342-x. 36002729

[ref9] Wachs S. J.; Behling C.; Ranninger J.; Möller J.; Mayrhofer K. J. J.; Berkes B. B. Online Monitoring of Transition-Metal Dissolution from a High-Ni-Content Cathode Material. ACS Appl. Mater. Interfaces 2021, 13, 33075–33082. 10.1021/acsami.1c07932. 34232020

[ref10] Wandt J.; Freiberg A.; Thomas R.; Gorlin Y.; Siebel A.; Jung R.; Gasteiger H. A.; Tromp M. Transition metal dissolution and deposition in Li-ion batteries investigated by operando X-ray absorption spectroscopy. Journal of Materials Chemistry A 2016, 4, 18300–18305. 10.1039/C6TA08865A.

[ref11] Ryu H.-H.; Namkoong B.; Kim J.-H.; Belharouak I.; Yoon C. S.; Sun Y.-K. Capacity Fading Mechanisms in Ni-Rich Single-Crystal NCM Cathodes. ACS Energy Letters 2021, 6, 2726–2734. 10.1021/acsenergylett.1c01089.

[ref12] Shi C.-G.; Peng X.; Dai P.; Xiao P.; Zheng W.-C.; Li H.-Y.; Li H.; Indris S.; Mangold S.; Hong Y.-H.; Luo C.-X.; Shen C.-H.; Wei Y.-M.; Huang L.; Sun S.-G. Investigation and Suppression of Oxygen Release by LiNi0.8Co0.1Mn0.1O2 Cathode under Overcharge Conditions. Adv. Energy Mater. 2022, 12, 2200569 10.1002/aenm.202200569.

[ref13] Geldasa F. T.; Kebede M. A.; Shura M. W.; Hone F. G. Identifying surface degradation, mechanical failure, and thermal instability phenomena of high energy density Ni-rich NCM cathode materials for lithium-ion batteries: a review. RSC Adv. 2022, 12, 5891–5909. 10.1039/D1RA08401A. 35424548 PMC8982025

[ref14] Park N.-Y.; Park G.-T.; Kim S.-B.; Jung W.; Park B.-C.; Sun Y.-K. Degradation Mechanism of Ni-Rich Cathode Materials: Focusing on Particle Interior. ACS Energy Letters 2022, 7, 2362–2369. 10.1021/acsenergylett.2c01272.

[ref15] Ruess R.; Schweidler S.; Hemmelmann H.; Conforto G.; Bielefeld A.; Weber D. A.; Sann J.; Elm M. T.; Janek J. Influence of NCM Particle Cracking on Kinetics of Lithium-Ion Batteries with Liquid or Solid Electrolyte. J. Electrochem. Soc. 2020, 167, 100532 10.1149/1945-7111/ab9a2c.

[ref16] Li W.; Liu X.; Xie Q.; You Y.; Chi M.; Manthiram A. Long-Term Cyclability of NCM-811 at High Voltages in Lithium-Ion Batteries: an In-Depth Diagnostic Study. Chem. Mater. 2020, 32, 7796–7804. 10.1021/acs.chemmater.0c02398.

[ref17] Ge S.; Longchamps R. S.; Liu T.; Liao J.; Leng Y.; Wang C.-Y. High safety and cycling stability of ultrahigh energy lithium ion batteries. Cell Reports Physical Science 2021, 2, 100584 10.1016/j.xcrp.2021.100584.

[ref18] Kim J.-M.; Zhang X.; Zhang J.-G.; Manthiram A.; Meng Y. S.; Xu W. A review on the stability and surface modification of layered transition-metal oxide cathodes. Mater. Today 2021, 46, 155–182. 10.1016/j.mattod.2020.12.017.

[ref19] Sun H. H.; Kim U.-H.; Park J.-H.; Park S.-W.; Seo D.-H.; Heller A.; Mullins C. B.; Yoon C. S.; Sun Y.-K. Transition metal-doped Ni-rich layered cathode materials for durable Li-ion batteries. Nat. Commun. 2021, 12, 6552 10.1038/s41467-021-26815-6. 34772958 PMC8589951

[ref20] Chen Z.; Nguyen H.-D.; Zarrabeitia M.; Liang H.-P.; Geiger D.; Kim J.-K.; Kaiser U.; Passerini S.; Iojoiu C.; Bresser D. Lithium Phosphonate Functionalized Polymer Coating for High-Energy Li[Ni0.8Co0.1Mn0.1]O2 with Superior Performance at Ambient and Elevated Temperatures. Adv. Funct. Mater. 2021, 31, 2105343 10.1002/adfm.202105343.

[ref21] Sim S.-J.; Lee S.-H.; Jin B.-S.; Kim H.-S. Use of carbon coating on LiNi0.8Co0.1Mn0.1O2 cathode material for enhanced performances of lithium-ion batteries. Sci. Rep. 2020, 10, 11114 10.1038/s41598-020-67818-5. 32632182 PMC7338463

[ref22] Li X.; Gu Q.; Qiu B.; Yin C.; Wei Z.; Wen W.; Zhang Y.; Zhou Y.; Gao H.; Liang H.; He Z.; Zhang M.; Meng Y. S.; Liu Z. Rational design of thermally stable polymorphic layered cathode materials for next generation lithium rechargeable batteries. Mater. Today 2022, 61, 91–103. 10.1016/j.mattod.2022.09.013.

[ref23] Zhao F.; Li X.; Yan Y.; Su M.; Liang L.; Nie P.; Hou L.; Chang L.; Yuan C. A three-in-one engineering strategy to achieve LiNi0.8Co0.1Mn0.1O2 cathodes with enhanced high-voltage cycle stability and high-rate capacities towards lithium storage. J. Power Sources 2022, 524, 231035 10.1016/j.jpowsour.2022.231035.

[ref24] Xue W.; Huang M.; Li Y.; Zhu Y. G.; Gao R.; Xiao X.; Zhang W.; Li S.; Xu G.; Yu Y.; Li P.; Lopez J.; Yu D.; Dong Y.; Fan W.; Shi Z.; Xiong R.; Sun C.-J.; Hwang I.; Lee W.-K.; Shao-Horn Y.; Johnson J. A.; Li J. Ultra-high-voltage Ni-rich layered cathodes in practical Li metal batteries enabled by a sulfonamide-based electrolyte. Nature Energy 2021, 6, 495–505. 10.1038/s41560-021-00792-y.

[ref25] Li X.; Liu J.; He J.; Wang H.; Qi S.; Wu D.; Huang J.; Li F.; Hu W.; Ma J. Hexafluoroisopropyl Trifluoromethanesulfonate-Driven Easily Li+ Desolvated Electrolyte to Afford Li||NCM811 Cells with Efficient Anode/Cathode Electrolyte Interphases. Adv. Funct. Mater. 2021, 31, 2104395 10.1002/adfm.202104395.

[ref26] Li Y.; Li W.; Shimizu R.; Cheng D.; Nguyen H.; Paulsen J.; Kumakura S.; Zhang M.; Meng Y. S. Elucidating the Effect of Borate Additive in High-Voltage Electrolyte for Li-Rich Layered Oxide Materials. Adv. Energy Mater. 2022, 12, 2103033 10.1002/aenm.202103033.

[ref27] Cho Y.-G.; Li M.; Holoubek J.; Li W.; Yin Y.; Meng Y. S.; Chen Z. Enabling the Low-Temperature Cycling of NMC||Graphite Pouch Cells with an Ester-Based Electrolyte. ACS Energy Letters 2021, 6, 2016–2023. 10.1021/acsenergylett.1c00484.

[ref28] Baird M. A.; Song J.; Tao R.; Ko Y.; Helms B. A. Locally Superconcentrated Electrolytes for Ultra-Fast-Charging Lithium Metal Batteries with High-Voltage Cathodes. ACS Energy Letters 2022, 7, 3826–3834. 10.1021/acsenergylett.2c02111.

[ref29] Li S.; Wang H.; Cuthbert J.; Liu T.; Whitacre J. F.; Matyjaszewski K. A Semiliquid Lithium Metal Anode. Joule 2019, 3, 1637–1646. 10.1016/j.joule.2019.05.022.

[ref30] Li S.; Mohamed A. I.; Pande V.; Wang H.; Cuthbert J.; Pan X.; He H.; Wang Z.; Viswanathan V.; Whitacre J. F.; Matyjaszewski K. Single-Ion Homopolymer Electrolytes with High Transference Number Prepared by Click Chemistry and Photoinduced Metal-Free Atom-Transfer Radical Polymerization. ACS Energy Letters 2018, 3, 20–27. 10.1021/acsenergylett.7b00999.

[ref31] Wang Y.; Wang Z.; Wu D.; Niu Q.; Lu P.; Ma T.; Su Y.; Chen L.; Li H.; Wu F. Stable Ni-rich layered oxide cathode for sulfide-based all-solid-state lithium battery. eScience 2022, 2, 537–545. 10.1016/j.esci.2022.06.001.

[ref32] Bi Y.; Li Q.; Yi R.; Xiao J. To Pave the Way for Large-Scale Electrode Processing of Moisture-Sensitive Ni-Rich Cathodes. J. Electrochem. Soc. 2022, 169, 020521 10.1149/1945-7111/ac4e5d.

[ref33] Narayan R.; Laberty-Robert C.; Pelta J.; Tarascon J.-M.; Dominko R. Self-Healing: An Emerging Technology for Next-Generation Smart Batteries. Adv. Energy Mater. 2022, 12, 2102652 10.1002/aenm.202102652.

[ref34] Dong T.; Mu P.; Zhang S.; Zhang H.; Liu W.; Cui G. How Do Polymer Binders Assist Transition Metal Oxide Cathodes to Address the Challenge of High-Voltage Lithium Battery Applications?. Electrochemical Energy Reviews 2021, 4, 545–565. 10.1007/s41918-021-00102-w.

[ref35] Chen H.; Ling M.; Hencz L.; Ling H. Y.; Li G.; Lin Z.; Liu G.; Zhang S. Exploring Chemical, Mechanical, and Electrical Functionalities of Binders for Advanced Energy-Storage Devices. Chem. Rev. 2018, 118, 8936–8982. 10.1021/acs.chemrev.8b00241. 30133259

[ref36] Zhen E.; Jiang J.; Lv C.; Huang X.; Xu H.; Dou H.; Zhang X. Effects of binder content on low-cost solvent-free electrodes made by dry-spraying manufacturing for lithium-ion batteries. J. Power Sources 2021, 515, 230644 10.1016/j.jpowsour.2021.230644.

[ref37] Hippauf F.; Schumm B.; Doerfler S.; Althues H.; Fujiki S.; Shiratsuchi T.; Tsujimura T.; Aihara Y.; Kaskel S. Overcoming binder limitations of sheet-type solid-state cathodes using a solvent-free dry-film approach. Energy Storage Materials 2019, 21, 390–398. 10.1016/j.ensm.2019.05.033.

[ref38] Li S.; Lorandi F.; Wang H.; Liu T.; Whitacre J. F.; Matyjaszewski K. Functional polymers for lithium metal batteries. Prog. Polym. Sci. 2021, 122, 101453 10.1016/j.progpolymsci.2021.101453.

[ref39] Saal A.; Hagemann T.; Schubert U. S. Polymers for Battery Applications—Active Materials, Membranes, and Binders. Adv. Energy Mater. 2021, 11, 2001984 10.1002/aenm.202001984.

[ref40] Ma Y.; Chen K.; Ma J.; Xu G.; Dong S.; Chen B.; Li J.; Chen Z.; Zhou X.; Cui G. A biomass based free radical scavenger binder endowing a compatible cathode interface for 5 V lithium-ion batteries. Energy Environ. Sci. 2019, 12, 273–280. 10.1039/C8EE02555J.

[ref41] Nguyen V. A.; Kuss C. Review—Conducting Polymer-Based Binders for Lithium-Ion Batteries and Beyond. J. Electrochem. Soc. 2020, 167, 065501 10.1149/1945-7111/ab856b.

[ref42] Chang B.; Kim J.; Cho Y.; Hwang I.; Jung M. S.; Char K.; Lee K. T.; Kim K. J.; Choi J. W. Highly Elastic Binder for Improved Cyclability of Nickel-Rich Layered Cathode Materials in Lithium-Ion Batteries. Adv. Energy Mater. 2020, 10, 2001069 10.1002/aenm.202001069.

[ref43] Ko S.; Baek M.-J.; Wi T.-U.; Kim J.; Park C.; Lim D.; Yeom S. J.; Bayramova K.; Lim H. Y.; Kwak S. K.; Lee S. W.; Jin S.; Lee D. W.; Lee H.-W. Understanding the Role of a Water-Soluble Catechol-Functionalized Binder for Silicon Anodes by Diverse In Situ Analyses. ACS Materials Letters 2022, 4, 831–839. 10.1021/acsmaterialslett.2c00013.

[ref44] Shi Y.; Zhou X.; Yu G. Material and Structural Design of Novel Binder Systems for High-Energy, High-Power Lithium-Ion Batteries. Acc. Chem. Res. 2017, 50, 2642–2652. 10.1021/acs.accounts.7b00402. 28981258

[ref45] Wang Y.; Dong N.; Liu B.; Qi K.; Tian G.; Qi S.; Wu D. Enhanced electrochemical performance of the LiNi0.8Co0.1Mn0.1O2 cathode via in-situ nanoscale surface modification with poly(imide-siloxane) binder. Chemical Engineering Journal 2022, 450, 137959 10.1016/j.cej.2022.137959.

[ref46] Qi K.; Wang Y.; Dong N.; Liu B.; Tian G.; Qi S.; Wu D. Novel polyimide binders integrated with soft and hard functional segments ensuring long-term high-voltage operating stability of high-energy NCM811 lithium-ion batteries up to 4.5 V. Applied Energy 2022, 320, 119282 10.1016/j.apenergy.2022.119282.

[ref47] Hong S.-B.; Lee Y.-J.; Kim U.-H.; Bak C.; Lee Y. M.; Cho W.; Hah H. J.; Sun Y.-K.; Kim D.-W. All-Solid-State Lithium Batteries: Li+-Conducting Ionomer Binder for Dry-Processed Composite Cathodes. ACS Energy Letters 2022, 7, 1092–1100. 10.1021/acsenergylett.1c02756.

[ref48] Wang Y.; Dong N.; Liu B.; Tian G.; Qi S.; Wu D. Self-adaptive Gel Poly(imide-siloxane) Binder Ensuring Stable Cathode-Electrolyte Interface for Achieving High-Performance NCM811 Cathode in Lithium-ion Batteries. Energy Storage Materials 2023, 56, 621–630. 10.1016/j.ensm.2023.02.002.

[ref49] Kim N.-Y.; Moon J.; Ryou M.-H.; Kim S.-H.; Kim J.-H.; Kim J.-M.; Bang J.; Lee S.-Y. Amphiphilic Bottlebrush Polymeric Binders for High-Mass-Loading Cathodes in Lithium-Ion Batteries. Adv. Energy Mater. 2022, 12, 2102109 10.1002/aenm.202102109.

[ref50] Liu Z.; Dong T.; Mu P.; Zhang H.; Liu W.; Cui G. Interfacial chemistry of vinylphenol-grafted PVDF binder ensuring compatible cathode interphase for lithium batteries. Chemical Engineering Journal 2022, 446, 136798 10.1016/j.cej.2022.136798.

[ref51] Han Y.; Heng S.; Wang Y.; Qu Q.; Zheng H. Anchoring Interfacial Nickel Cations on Single-Crystal LiNi0.8Co0.1Mn0.1O2 Cathode Surface via Controllable Electron Transfer. ACS Energy Letters 2020, 5, 2421–2433. 10.1021/acsenergylett.0c01032.

[ref52] Zheng M.; Fu X.; Wang Y.; Reeve J.; Scudiero L.; Zhong W.-H. Poly(Vinylidene Fluoride)-Based Blends as New Binders for Lithium-Ion Batteries. ChemElectroChem. 2018, 5, 2288–2294. 10.1002/celc.201800553.

[ref53] Hester J. F.; Banerjee P.; Won Y. Y.; Akthakul A.; Acar M. H.; Mayes A. M. ATRP of Amphiphilic Graft Copolymers Based on PVDF and Their Use as Membrane Additives. Macromolecules 2002, 35, 7652–7661. 10.1021/ma0122270.

[ref54] Shen L.; Feng S.; Li J.; Chen J.; Li F.; Lin H.; Yu G. Surface modification of polyvinylidene fluoride (PVDF) membrane via radiation grafting: novel mechanisms underlying the interesting enhanced membrane performance. Sci. Rep. 2017, 7, 2721 10.1038/s41598-017-02605-3. 28578428 PMC5457412

[ref55] Lanzalaco S.; Fantin M.; Scialdone O.; Galia A.; Isse A. A.; Gennaro A.; Matyjaszewski K. Atom Transfer Radical Polymerization with Different Halides (F, Cl, Br, and I): Is the Process “Living” in the Presence of Fluorinated Initiators?. Macromolecules 2017, 50, 192–202. 10.1021/acs.macromol.6b02286.

[ref56] Zhang M.; Russell T. P. Graft Copolymers from Poly(vinylidene fluoride-co-chlorotrifluoroethylene) via Atom Transfer Radical Polymerization. Macromolecules 2006, 39, 3531–3539. 10.1021/ma060128m.

[ref57] Matyjaszewski K. Atom Transfer Radical Polymerization (ATRP): Current Status and Future Perspectives. Macromolecules 2012, 45, 4015–4039. 10.1021/ma3001719.

[ref58] Matyjaszewski K.; Tsarevsky N. V. Macromolecular Engineering by Atom Transfer Radical Polymerization. J. Am. Chem. Soc. 2014, 136, 6513–6533. 10.1021/ja408069v. 24758377

[ref59] Liu T.; Wu X.; Zhu S.; Lorandi F.; Ni L.; Li S.; Sun M.; Bloom B. P.; Waldeck D. H.; Viswanathan V.; Whitacre J. F.; Matyjaszewski K. Polymer-Stabilized Liquid Metal Nanoparticles as a Scalable Current Collector Engineering Approach Enabling Lithium Metal Anodes. ACS Applied Energy Materials 2022, 5, 3615–3625. 10.1021/acsaem.1c04106.

[ref60] Matyjaszewski K.; Tsarevsky N. V. Nanostructured functional materials prepared by atom transfer radical polymerization. Nat. Chem. 2009, 1, 276–288. 10.1038/nchem.257. 21378870

[ref61] Matyjaszewski K. Architecturally Complex Polymers with Controlled Heterogeneity. Science 2011, 333, 1104–1105. 10.1126/science.1209660. 21868664

[ref62] Li S.; Liu T.; Yan J.; Flum J.; Wang H.; Lorandi F.; Wang Z.; Fu L.; Hu L.; Zhao Y.; Yuan R.; Sun M.; Whitacre J. F.; Matyjaszewski K. Grafting polymer from oxygen-vacancy-rich nanoparticles to enable protective layers for stable lithium metal anode. Nano Energy 2020, 76, 105046 10.1016/j.nanoen.2020.105046.

[ref63] Corrigan N.; Jung K.; Moad G.; Hawker C. J.; Matyjaszewski K.; Boyer C. Reversible-deactivation radical polymerization (Controlled/living radical polymerization): From discovery to materials design and applications. Prog. Polym. Sci. 2020, 111, 101311 10.1016/j.progpolymsci.2020.101311.

[ref64] Szczepaniak G.; Jeong J.; Kapil K.; Dadashi-Silab S.; Yerneni S. S.; Ratajczyk P.; Lathwal S.; Schild D. J.; Das S. R.; Matyjaszewski K. Open-air green-light-driven ATRP enabled by dual photoredox/copper catalysis. Chemical Science 2022, 13, 11540–11550. 10.1039/D2SC04210J. 36320395 PMC9557244

[ref65] Matyjaszewski K. Advanced Materials by Atom Transfer Radical Polymerization. Adv. Mater. 2018, 30, 1706441 10.1002/adma.201706441. 29582478

[ref66] Xiong J.; Dupré N.; Mazouzi D.; Guyomard D.; Roué L.; Lestriez B. Influence of the Polyacrylic Acid Binder Neutralization Degree on the Initial Electrochemical Behavior of a Silicon/Graphite Electrode. ACS Appl. Mater. Interfaces 2021, 13, 28304–28323. 10.1021/acsami.1c06683. 34101424

[ref67] Ahmed S.; Pokle A.; Schweidler S.; Beyer A.; Bianchini M.; Walther F.; Mazilkin A.; Hartmann P.; Brezesinski T.; Janek J.; Volz K. The Role of Intragranular Nanopores in Capacity Fade of Nickel-Rich Layered Li(Ni1-x-yCoxMny)O2 Cathode Materials. ACS Nano 2019, 13, 10694–10704. 10.1021/acsnano.9b05047. 31480835

[ref68] Zhang Z.; Yang J.; Huang W.; Wang H.; Zhou W.; Li Y.; Li Y.; Xu J.; Huang W.; Chiu W.; Cui Y. Cathode-Electrolyte Interphase in Lithium Batteries Revealed by Cryogenic Electron Microscopy. Matter 2021, 4, 302–312. 10.1016/j.matt.2020.10.021.

[ref69] Weng S.; Li Y.; Wang X. Cryo-EM for battery materials and interfaces: Workflow, achievements, and perspectives. iScience 2021, 24, 103402 10.1016/j.isci.2021.103402. 34849466 PMC8607198

[ref70] Sahore R.; O’Hanlon D. C.; Tornheim A.; Lee C.-W.; Garcia J. C.; Iddir H.; Balasubramanian M.; Bloom I. Revisiting the Mechanism Behind Transition-Metal Dissolution from Delithiated LiNixMnyCozO2 (NMC) Cathodes. J. Electrochem. Soc. 2020, 167, 020513 10.1149/1945-7111/ab6826.

[ref71] Jung R.; Linsenmann F.; Thomas R.; Wandt J.; Solchenbach S.; Maglia F.; Stinner C.; Tromp M.; Gasteiger H. A. Nickel, Manganese, and Cobalt Dissolution from Ni-Rich NMC and Their Effects on NMC622-Graphite Cells. J. Electrochem. Soc. 2019, 166, A378 10.1149/2.1151902jes.

[ref72] Gao S.; Zhan X.; Cheng Y.-T. Structural, electrochemical and Li-ion transport properties of Zr-modified LiNi0.8Co0.1Mn0.1O2 positive electrode materials for Li-ion batteries. J. Power Sources 2019, 410–411, 45–52. 10.1016/j.jpowsour.2018.10.094.

[ref73] Hong C.; Leng Q.; Zhu J.; Zheng S.; He H.; Li Y.; Liu R.; Wan J.; Yang Y. Revealing the correlation between structural evolution and Li+ diffusion kinetics of nickel-rich cathode materials in Li-ion batteries. Journal of Materials Chemistry A 2020, 8, 8540–8547. 10.1039/D0TA00555J.

[ref74] Phillip N. D.; Daniel C.; Veith G. M. Influence of Binder Coverage on Interfacial Chemistry of Thin Film LiNi0.6Mn0.2Co0.2O2 Cathodes. J. Electrochem. Soc. 2020, 167, 040521 10.1149/1945-7111/ab78fc.

[ref75] Park S.; Jeong S. Y.; Lee T. K.; Park M. W.; Lim H. Y.; Sung J.; Cho J.; Kwak S. K.; Hong S. Y.; Choi N.-S. Replacing conventional battery electrolyte additives with dioxolone derivatives for high-energy-density lithium-ion batteries. Nat. Commun. 2021, 12, 838 10.1038/s41467-021-21106-6. 33547320 PMC7864909

[ref76] Gao H.; Cai J.; Xu G.-L.; Li L.; Ren Y.; Meng X.; Amine K.; Chen Z. Surface Modification for Suppressing Interfacial Parasitic Reactions of a Nickel-Rich Lithium-Ion Cathode. Chem. Mater. 2019, 31, 2723–2730. 10.1021/acs.chemmater.8b04200.

[ref77] Li D.; Li H.; Danilov D. L.; Gao L.; Chen X.; Zhang Z.; Zhou J.; Eichel R.-A.; Yang Y.; Notten P. H. L. Degradation mechanisms of C6/LiNi0.5Mn0.3Co0.2O2 Li-ion batteries unraveled by non-destructive and post-mortem methods. J. Power Sources 2019, 416, 163–174. 10.1016/j.jpowsour.2019.01.083.

[ref78] Biesinger M. C.; Lau L. W. M.; Gerson A. R.; Smart R. S. C. The role of the Auger parameter in XPS studies of nickel metal, halides and oxides. Phys. Chem. Chem. Phys. 2012, 14, 2434–2442. 10.1039/c2cp22419d. 22249653

[ref79] Davidson A.; Tempere J. F.; Che M.; Roulet H.; Dufour G. Spectroscopic Studies of Nickel(II) and Nickel(III) Species Generated upon Thermal Treatments of Nickel/Ceria-Supported Materials. J. Phys. Chem. 1996, 100, 4919–4929. 10.1021/jp952268w.

[ref80] Zheng J.; Ye Y.; Liu T.; Xiao Y.; Wang C.; Wang F.; Pan F. Ni/Li Disordering in Layered Transition Metal Oxide: Electrochemical Impact, Origin, and Control. Acc. Chem. Res. 2019, 52, 2201–2209. 10.1021/acs.accounts.9b00033. 31180201

