# Supplementary material for: Tailored PVDF Graft Copolymers via ATRP as High-Performance NCM811 Cathode Binders
Source: ACS Mater Lett. 2023 Aug 25;5(10):2594–603. doi: 10.1021/acsmaterialslett.3c00485 (PMC10548467; doi:10.1021/acsmaterialslett.3c00485)
Supplement: Supplementary file 1 — tz3c00485_si_001.pdf [file tz3c00485_si_001.pdf]

Supplementary Information

Tailored PVDF Graft Copolymers *via* ATRP as High-Performance NCM811 Cathode Binders

Tong Liu, [a,1] *Rohan Parekh,* [a,b,1] *Piotr Mocny,* [a] *Brian P. Bloom,* [c] *Yuqi Zhao,* [b] *So Young An,* [a] *Bonian Pan,*[b] *Rongguan Yin,*[a] *David H. Waldeck,*[c] *Jay F. Whitacre,**[b,d] *and Krzysztof Matyjaszewski**[a]

---

[a] Department of Chemistry, Carnegie Mellon University, 4400 Fifth Ave. Pittsburgh, PA, 15213

[b] Department of Materials Science and Engineering, Carnegie Mellon University, 5000 Forbes Ave. Pittsburgh, PA, 15213

[c] Department of Chemistry, University of Pittsburgh, 219 Parkman Avenue, Pittsburgh, PA, 15260

[d] Scott Institute for Energy Innovation, Carnegie Mellon University, 5000 Forbes Ave. Pittsburgh, PA, 15213

* Corresponding authors

Jay F. Whitacre whitacre@andrew.cmu.edu

Krzysztof Matyjaszewski matyjaszewski@cmu.edu

[1] These authors contributed equally to this work.

Materials and Characterizations.

Materials *tert*-Butyl acrylate (*t*BA, Sigma-Aldrich, >99%), poly(ethylene glycol) methyl ether acrylate (OEGA, M_n = 480 g/mol, Sigma-Aldrich, >99%) were purified by passing through a basic alumina column before use. Tris(2-dimethylaminoethyl)amine (Me_6TREN, Ambeed, 99%), $CuCl_2$ (Sigma-Aldrich, 99%), Eosin Y disodium salt (Sigma-Aldrich), poly(vinylidene fluoride) (PVDF, Sigma-Aldrich), poly(vinylidene fluoride-co-chlorotrifluoroethylene) (PVDF-CTFE 90/10 wt%, PolyK Technologies), trifluoroacetic acid (TFA, >99%, Sigma-Aldrich), diethyl ether (Fisher Chemical, 99%), methanol (Fisher Chemical, 99.8%), *N*,*N*-dimethylformamide (DMF, VWR, 99%), 1-methyl-2-pyrrolidinone (NMP, Sigma Aldrich, anhydrous, 99.5%), dimethyl sulfoxide (DMSO, Fisher Chemical, 99.5%) were used as received.

Nuclear Magnetic Resonance Spectroscopy (NMR). ^{1}H NMR spectroscopy measurements were performed on a Bruker Advance 300 MHz spectrometer using DMSO-d6 as the solvent.

Gel Permeation Chromatography (GPC). Number-average molecular weights (M_n) and molecular weight distributions (MWD) of the polymers were determined by GPC. The THF GPC system used an Agilent 1260 infinity II pump, a Waters 2414 refractive index detector, a PSS SLD9000 MALS detector, PSS columns (SDV 10^3, 10^5, and 10^6 Å) with THF as the eluent at a flow rate of 1 mL/min at 35 °C. The DMF GPC system used an Agilent 1260 infinity II pump, a PSS SLD2020 MALS detector, PSS columns (GRAM 100, 3000, 3000 Å) with DMF as an eluent at 50 °C and the flow rate of 1 mL/min. To obtain absolute molecular weight, dn/dc values were measured by preparing GPC samples with different concentrations of each polymer (1 mg/mL, 2 mg/mL, 3 mg/mL, 4 mg/mL, and 5 mg/mL). Then the injected mass was plotted vs. area. The dn/dc was determined from the slope, which reflects the detector constant multiplied by the dn/dc. The obtained dn/dc values were then used for the calculation of the absolute molecular weight of each polymer.

Fourier-transform infrared spectroscopy (FTIR). FTIR was carried out using a PerkinElmer Frontier spectrometer with a germanium crystal exploring a 1 cm^{-1} of resolution over a range of 700–4000 cm^{-1}.

Thermogravimetric Analysis (TGA). TGA with TA Instruments 2950 was used to measure the thermal stability of the polymers under nitrogen. The data were analyzed with TA Universal

Analysis. The heating procedure involved four steps: (1) temperature ramp up to 110 ºC at a rate of 30 ºC/min; (2) hold at 110 ºC for 10 min; (3) ramp up at a rate of 30 ºC/min to 800 ºC; (4) hold for 3 min.

Differential Scanning Calorimetry (DSC). The glass transition temperature (T_g) of PVDF, PVDF-CTFE, and PVDF-CTFE-*g*-PEGA-co-PAA were measured by DSC with TA Instrument QA-2000. The samples were first equilibrated at 25.00 °C and isothermal for 1.00 min, following three cooling/heating cycles, each involving the following steps: (1) Ramp 10.00 °C/min to -90.00 °C, (2) Isothermal for 1.00 min, (3) Ramp 10.00 °C/min to 200.00 °C, (4) Isothermal for 1.00 min. The DSC data were analyzed with a TA Universal Analysis instrument, and T_g were acquired directly.

Dynamic mechanical analysis (DMA): Bulk film fabrication. The PVDF, PVDF-CTFE, and PVDF-CTFE-*g*-PEGA-co-PAA polymer solutions in NMP (8 wt%) were drop-casted into 15 mm × 5 mm rectangular Teflon molds. After most of the solvent was slowly evaporated over 24 h at room temperature, molds were transferred to a vacuum oven and continuously evaporated at 120 °C for 48 hours. Transparent free-standing films with thicknesses of 100-200 μm were obtained.

Tensile test. The bulk films were tested in the tensile mode by using DMA (TA RSA-G2). The film thickness was between 100-200 μm. The samples were stretched at a constant tensile rate of 0.05 mm/mm · s at room temperature.

Peel test. 1 cm-wide Scotch tape (3M 600) was first firmly pressed and adhered to NCM811 cathodes on Al foil prepared with PVDF, PVDF-CTFE and PVDF-CTFE-*g*-PEGA-co-PAA binders, respectively. One end of the electrode was clamped and fixed on the bottom geometry of the DMA instrument, and one end of the tape was clamped on the top geometry that can move freely. Then the tape was peeled at an angle of 180 degrees from the electrode at a constant peeling rate of 0.5 mm/s, the adhesion strength of the electrodes was recorded as the force of the geometry (N).

Scanning electron microscope (SEM). SEM was performed for surface morphology investigation and conducted on a Quanta 600 FEG instrument. Images were acquired through ETD detector at 30 kV with spot size 3.5.

Focused Ion Beam Scanning Electron Microscope (FIBSEM). FIBSEM was conducted on FEI Helios Xenon PFIB. An accelerating voltage of 5 kV and beam current of 0.34 nA were used for imaging. A plasma beam current of 6.7 nA and 1.8 nA were used for particle milling. 1-1.5 μm of Pt was coated on the samples before ion-milling to enable the clear and fine cross-section images.

Micro Computed Tomography (Micro-CT). Each electrode was cut into rectangular shape with a width of around 1 mm and height of 5 mm, then was mounted on a sample holder using fast-setting epoxy and placed into an Xradia Crystal CT (Zeiss Group). By extending the detector and moving Xray source close to their limits, a Voxel size of 0.74 μm was achieved. The scan was performed with X-ray source set to 40 kV, 3 A, air filter, bin 1, and 0.5s x 5 exposures per projection using Scout and Scan Control System (Zeiss Group). Reconstruction was done manually to account for large centering shift using Reconstructor Scout-and-Scan Control System program (Zeiss Group). The reconstructed .txm file was then processed with Dragonfly software (Object Research System). After cropping each sample into 500 μm by 500 μm by electrode height blocks where the images are least distorted and contains only electrodes, they were partitioned into pore space and non-pore space based on the X-ray signal strength of each individual voxel using Dragonfly's Otsu thresholding tool. A threshold of 31000 unit was used on all three samples to ensure consistency and comparability between the samples.

X-ray photoelectron spectroscopy (XPS). The XPS spectra were collected using a Thermo Fisher ESCALAB 250 Xi instrument with a 650 μm acquisition spot size. A flood gun was used during data collection and each spectrum was referenced to the adventitious carbon C-C peak at 284.8 eV. Each plot represents the average of at least 10 scans and the spectra were fit using Avantage software package.

X-ray diffraction (XRD). Powder XRD was conducted between 2θ values of 10° and 80° with Cu Kα radiation (1.5406 Å) using a Malvern Panalytical Empyrean X-ray diffractometer with 1Der detector.

Inductively coupled plasma mass spectrometry (ICP-MS). After disassembly of the cycled Li|NCM811 cells, all the other parts except NCM811 cathodes were collected and harvested from the cell, then soaked in 2 mL dimethyl carbonate for 24h. 100 μL of the solution were

taken and added into 0.5 mL 70% HNO_3 for digestion. The digested samples were further diluted to 3.5% HNO_3 by ultra-purified water and filtered by a 0.45 μm PTFE syringe filter before analysis by ICP-MS. (Agilent 7700X).

Synthesis of PVDF-CTFE-*g*-PEGA-co-P*t*BA

In a 20 mL vial, 1.2 g (0.00103 mol) PVDF-CTFE was dissolved in 8 mL DMF and 1 mL DMSO. Then 1.13 mL (1.24 g, 0.0026 mol) of OEGA480, 2.64 mL (2.31 g, 0.018 mol) of *t*BA, 110.8 μL (0.00412 mmol, 5 mg/mL solution in DMF) of $CuCl_2$, 95 μL (0.412 μmol, 3 mg/mL solution in DMSO) of Eosin Y disodium salt and 142.4 μL (0.0124 mmol, 20 mg/mL solution in DMF) of Me_6TREN were added to the vial. The vial was then covered with a rubber stopper and the reaction mixture was degassed by nitrogen bubbling for 5 minutes. It was then placed under a green light lamp at room temperature. Once the reaction was complete with the target conversion monitored by ^{1}H NMR, the reaction mixture was precipitated in 50 vol% methanol/water, redissolved in acetone, and precipitated two more times. The solid product was then dried and collected.

Hydrolysis of PVDF-CTFE-*g*-PEGA-co-P*t*BA

The dry product of PVDF-CTFE-*g*-PEGA-co-P*t*BA polymer (1.63 g) and 0.2 mL TFA were added to a Schlenk flask and sealed under vacuum, then left in vacuum oven overnight at 60°C. The resultant polymer was then washed with DCM and analyzed using ^{1}H NMR to monitor the completion of conversion of *tert*-butyl groups to -COOH group, after drying under vacuum at 50°C overnight, the PVDF-CTFE-*g*-PEGA-co-PAA product was obtained and used directly as binder for NCM811 cathode preparation.

Cell assembly and electrochemical testing

Electrochemical characterization was carried out using 2032-type coin cells with lithium metal anodes, celgard battery separator, 0.5 mm stainless steel spacer, and wave spring. 1M lithium hexafluorophosphate (LiPF6) in a mixture solution of ethylene carbonate (EC) and ethyl methyl carbonate (EMC) (3:7 v/v) was used as the liquid electrolyte. NCM811 cathodes were prepared with composition of NCM811/carbon black/polymer binders = 85/10/5 and 93/4/3 wt%. The well-mixed cathode slurry was cast onto Al foil (active material loading ~ 5 mg/cm^2) and vacuum dried for 8h at ~ 90°C. All Li|NCM811 coin cells were assembled using 600 μm thick Li metal, as-prepared NCM811 cathodes and 60 μL of liquid electrolyte to standardize the testing. All the cells were assembled in a glove box with water/oxygen content lower than

0.5 ppm. Rate tests and long-term testing of Li|NCM811 cell were conducted on Landt Instruments at room temperature. For *post-mortem* analysis of cycled NCM811 cathodes, coin cells were disassembled in glove box, then NCM811 cathodes were washed by dimethyl carbonate (DMC). After vacuum drying, the cycled cathodes were directly used for XPS, SEM and FIB-SEM analysis.

Table S1. Samples of grafted PVDF-CTFE polymers and their performance as binders in Li|NCM811 cells.

Entry	Sample	[M]/[CI]	Grafted polymers (wt%)	Molecular weight [a]	Dispersity [a]	Discharge capacity at 0.1, 0.2, 0.5, 1, 2, and 4C (mAh/g) [c]
**1**	PVDF	N/A	0	107,000	2.57	202.6, 195, 184.9, 172.3, 156.5, 103.8
**2**	PVDF-CTFE	N/A	0	108,000	2.70	205.2, 196.5, 186, 173.5, 158.4, 109.1
**3**[b]	PVDF-CTFE-*g*-P*t*BA	10	20	335,000	2.00	155, 136.7, 116.7, 99.1, N/A, N/A
**4**[b]	PVDF-CTFE-*g*-P*t*BA	80	70	323,000	1.96	177.5, 168.3, 122.3, 69.0, N/A, N/A
**5**	PVDF-CTFE-*g*-PEGA	40	89	685,000	1.48	N/A
**6**	PVDF-CTFE-*g*-PEGA	5	20	280,000	2.64	203.8, 194.6, 183.5, 171.4, 155.6, 112.2
**7**[b]	PVDF-CTFE-*g*-PEGA-co-P*t*BA	OEGA-2.5<br>tBA-17.5	PEGA-10<br>PtBA-10	354,000	2.74	204.3, 197, 184.8, 174.4, 160.6, 128.7

[a] Absolute molecular weight determined by DMF GPC with MALS detector.

[b] Absolute molecular weight determined by THF GPC with MALS detector. PVDF-CTFE-*g*-PEGA-co-P*t*BA were further exposed to TFA to obtain PVDF-CTFE-*g*-PEGA-co-PAA to be used as binders for NCM811 cathodes.

[c] Mean discharge capacities at different C-rates were reported from Li|NCM811 cells in the voltage range of 2.9-4.3V using NCM811 cathodes (NCM811/carbon black/binder = 85/10/5 wt%) prepared by corresponding polymer binders to select optimal binder composition.

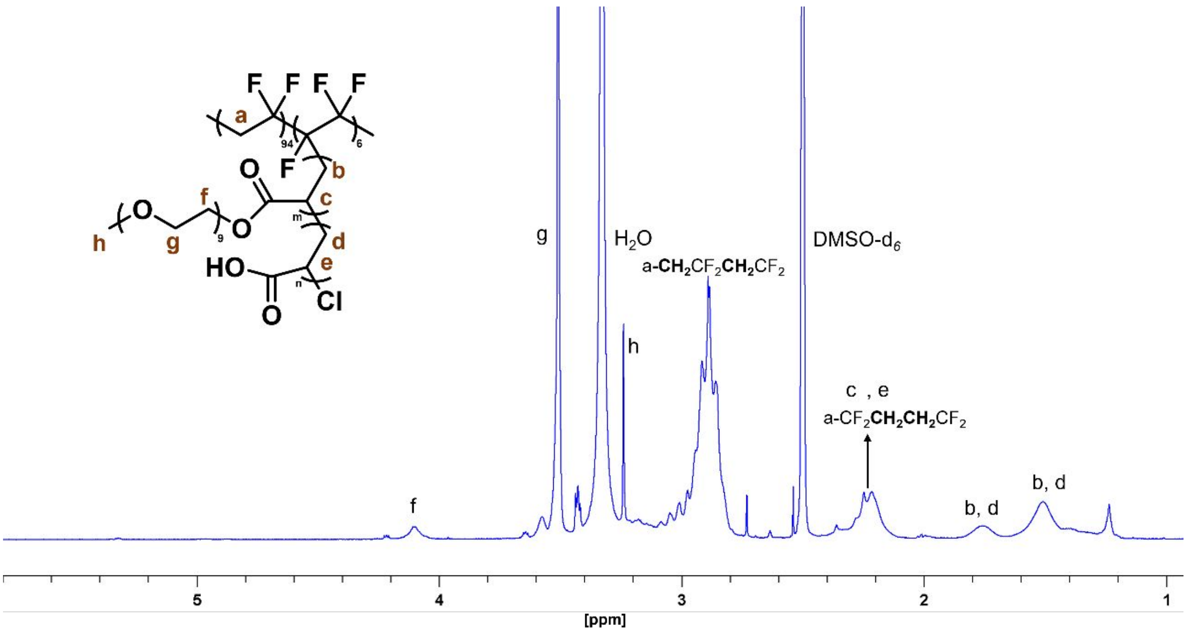

Figure S1. ^{1}HNMR spectrum of PVDF-CTFE-*g*-PEGA-co-PAA.

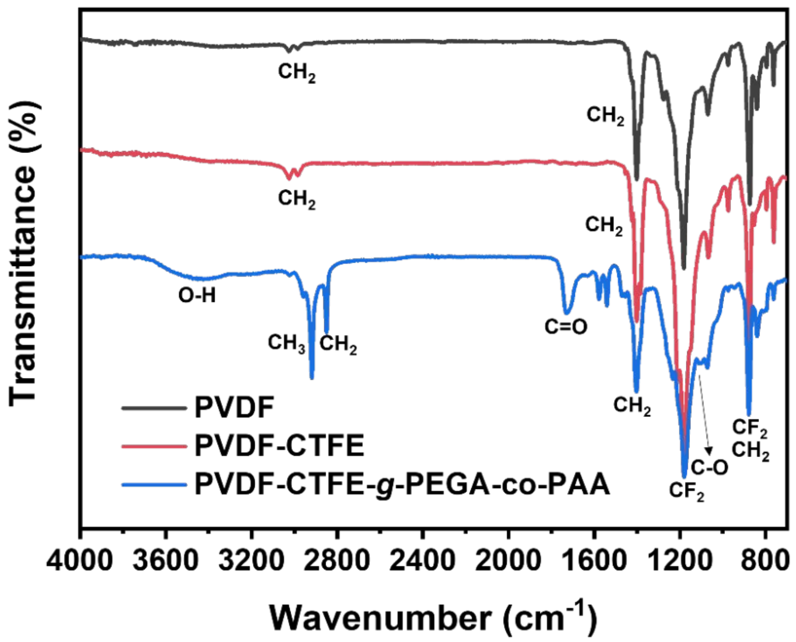

Figure S2. FTIR of PVDF (dark grey), PVDF-CTFE (red) and PVDF-CTFE-*g*-PEGA-co-PAA (blue).

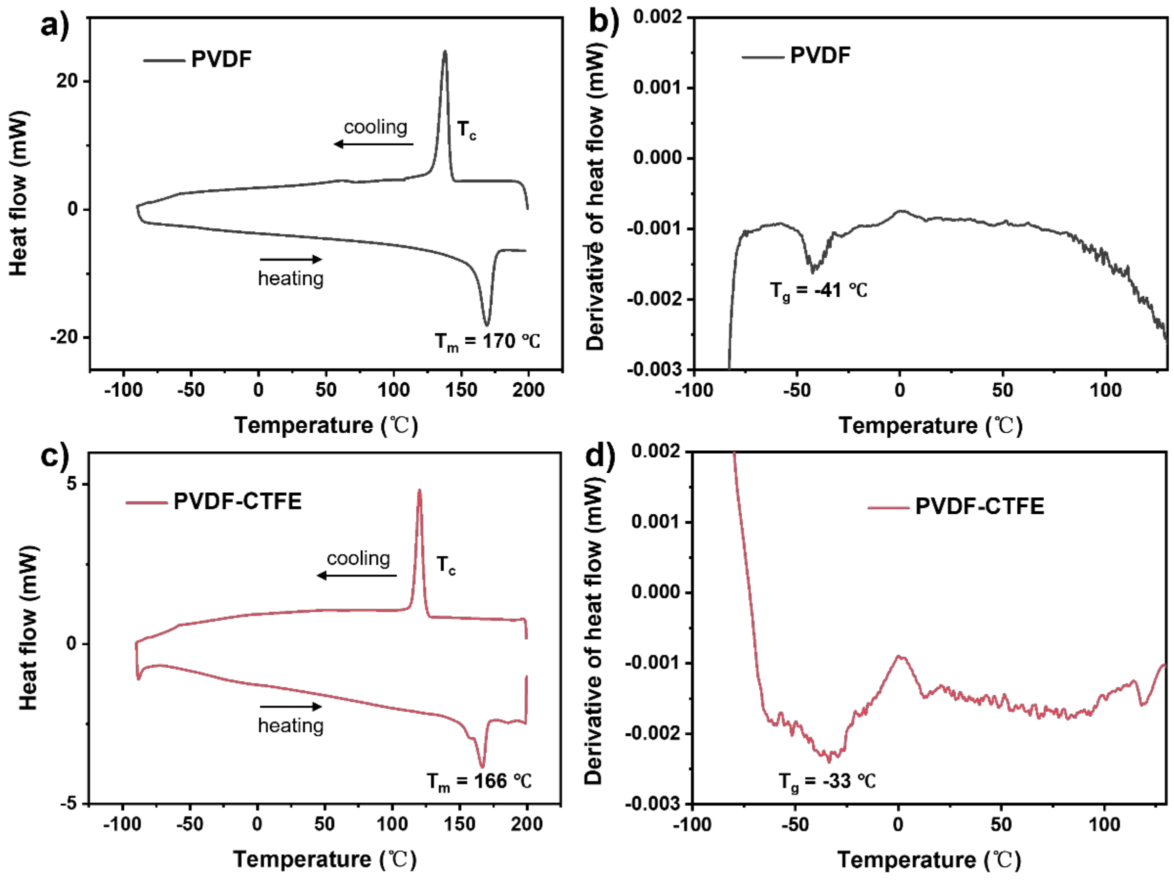

Figure S3. DSC curves of a) PVDF; b) derivative of DSC of PVDF; c) DSC curves of PVDF-CTFE; d) derivative of DSC of PVDF-CTFE.

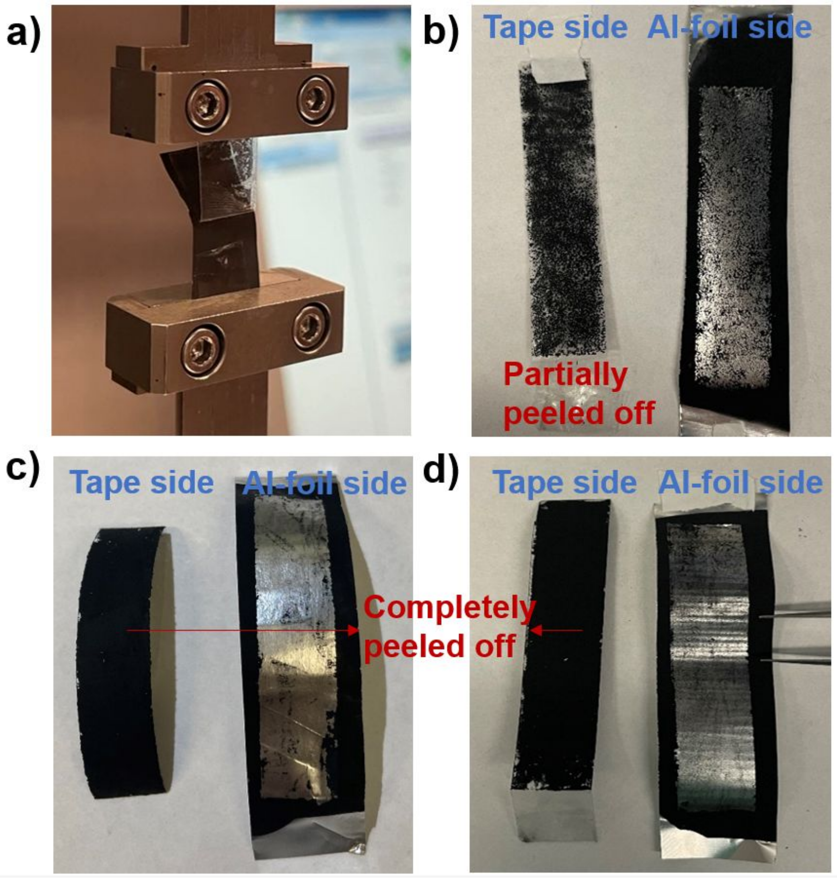

Figure S4. Photographs of a) 180° peel test set up. NCM811 cathodes on adhesive tape and Al substrate after peel test with b) PVDF c) PVDF-CTFE and d) PVDF-CTFE-*g*-PEGA-co-PAA binder.

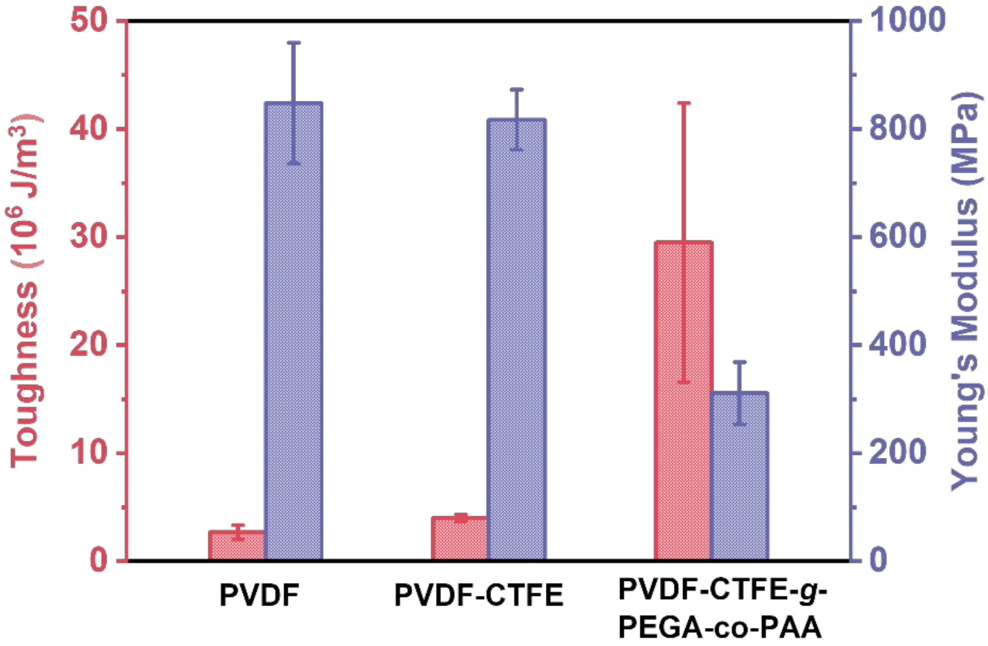

Figure S5. Toughness and Young's modulus calculated from stress-strain test of PVDF, PVDF-CTFE and PVDF-CTFE-*g*-PEGA-co-PAA.

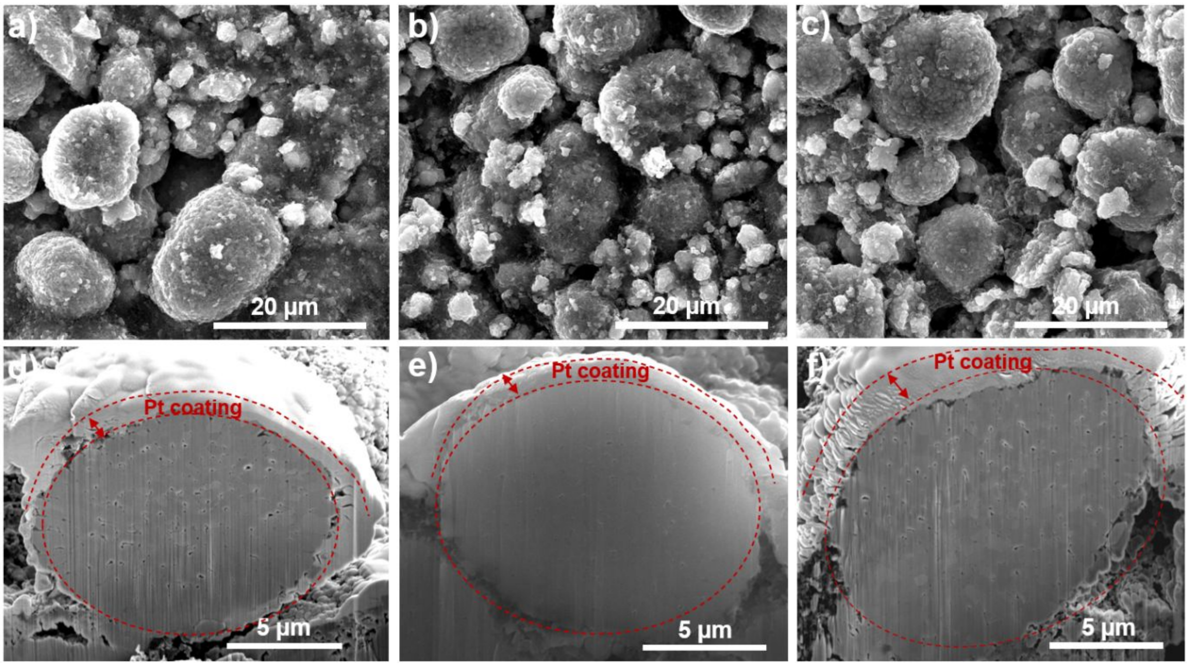

Figure S6. Top-view SEM images of NCM811 cathodes before cycling with a) PVDF, b) PVDF-CTFE, and c) PVDF-CTFE-*g*-PEGA-co-PAA binder. FIB-SEM of cross-section of NCM811 cathodes before cycling with d) PVDF, e) PVDF-CTFE, and f) PVDF-CTFE-*g*-PEGA-co-PAA binder.

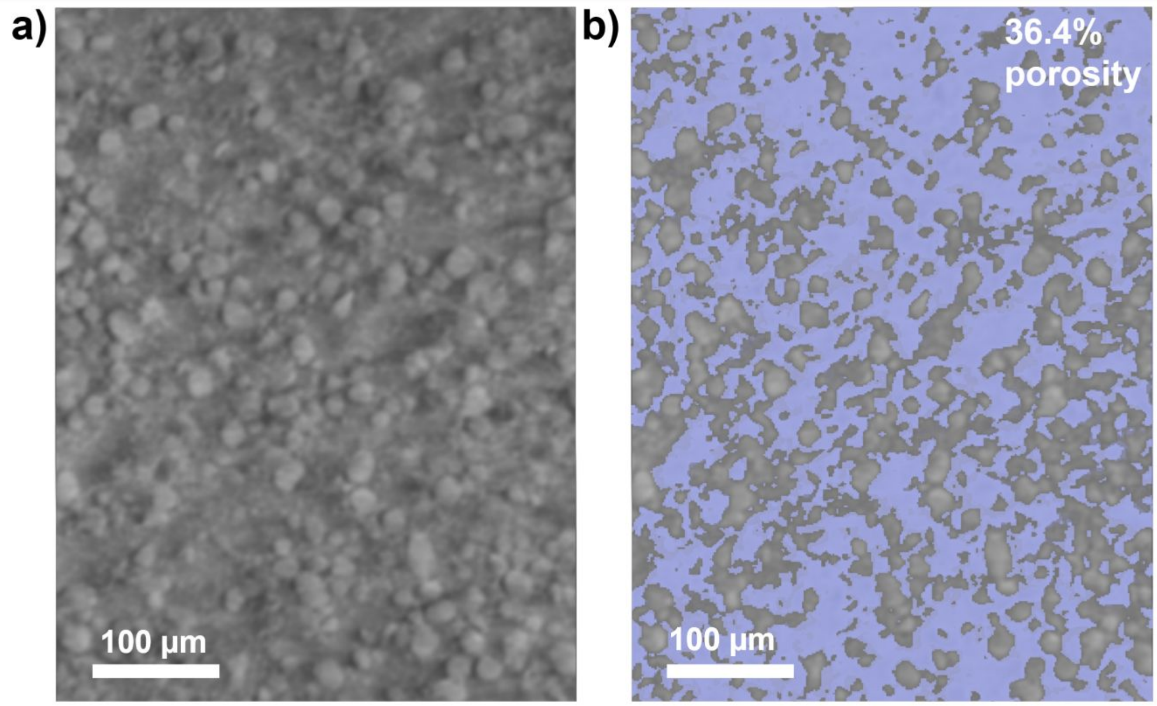

Figure S7. Micro CT of NCM811 cathodes with PVDF binder. a) The raw data of the reconstructed gray-scale image; b) thresholding segmentation of the active material, binder, and carbon black. Porosity: 36.4%.

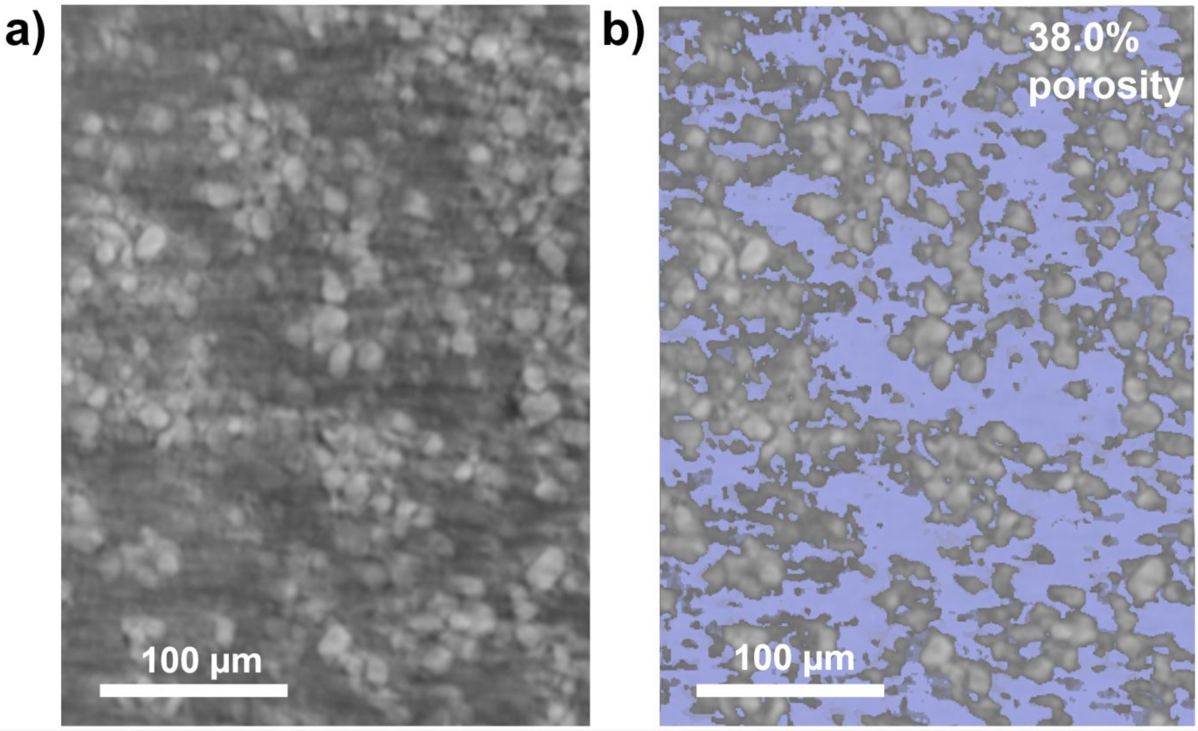

Figure S8. Micro CT of NCM811 cathodes with PVDF-CTFE binder. a) The raw data of the reconstructed gray-scale image; b) thresholding segmentation of the active material, binder, and carbon black. Porosity: 38.0%.

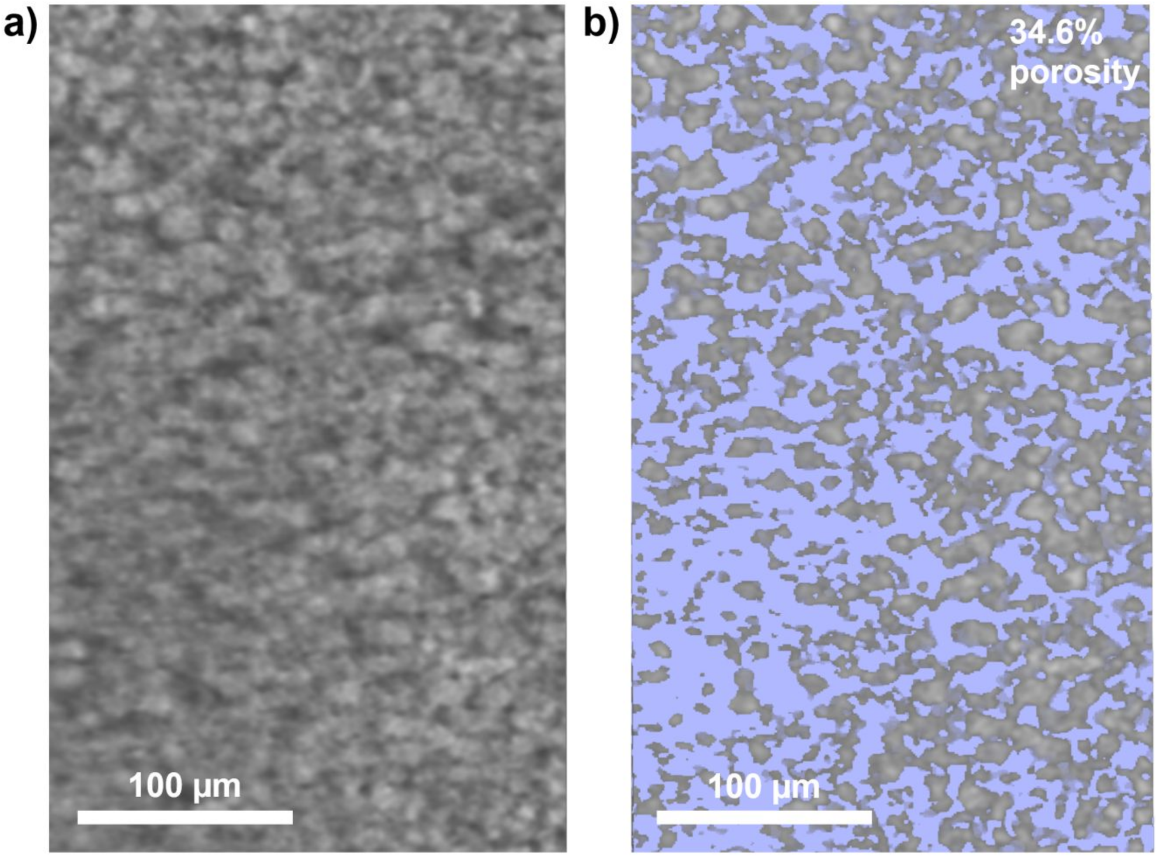

Figure S9. Micro CT of NCM811 cathodes with PVDF-CTFE-*g*-PEGA-co-PAA binder. a) The raw data of the reconstructed gray-scale image; b) thresholding segmentation of the active material, binder, and carbon black. Porosity: 34.6%.

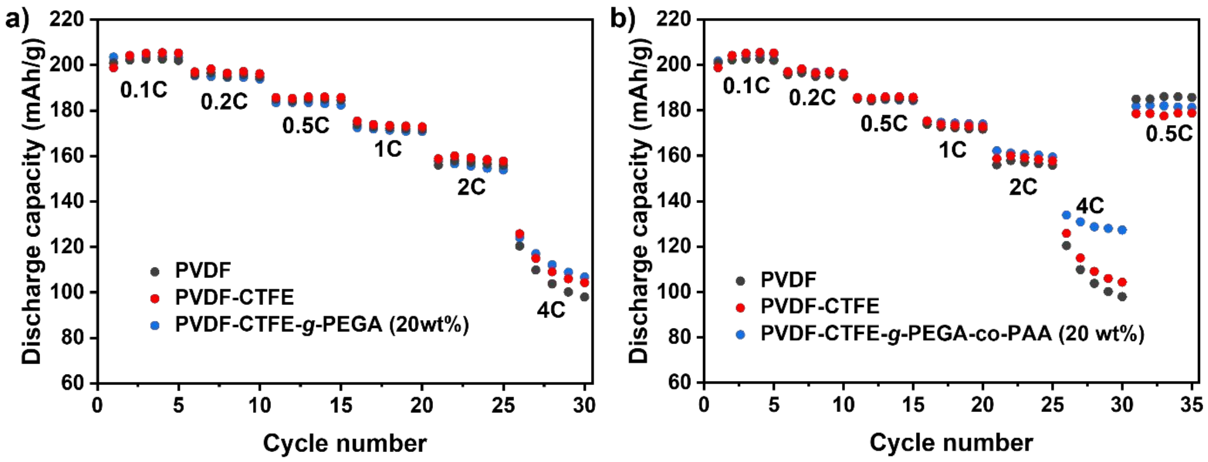

Figure S10. Rate performance of Li|NCM811 cells with composition of NCM811/carbon black/binder = 85/10/5 (wt%) using a) PVDF-CTFE-*g*-PEGA20, b) PVDF-CTFE-*g*-PEGA10-co-PAA10 binder with PVDF (dark grey) and PVDF-CTFE (red) as comparison binders in the voltage range 2.9-4.3V.

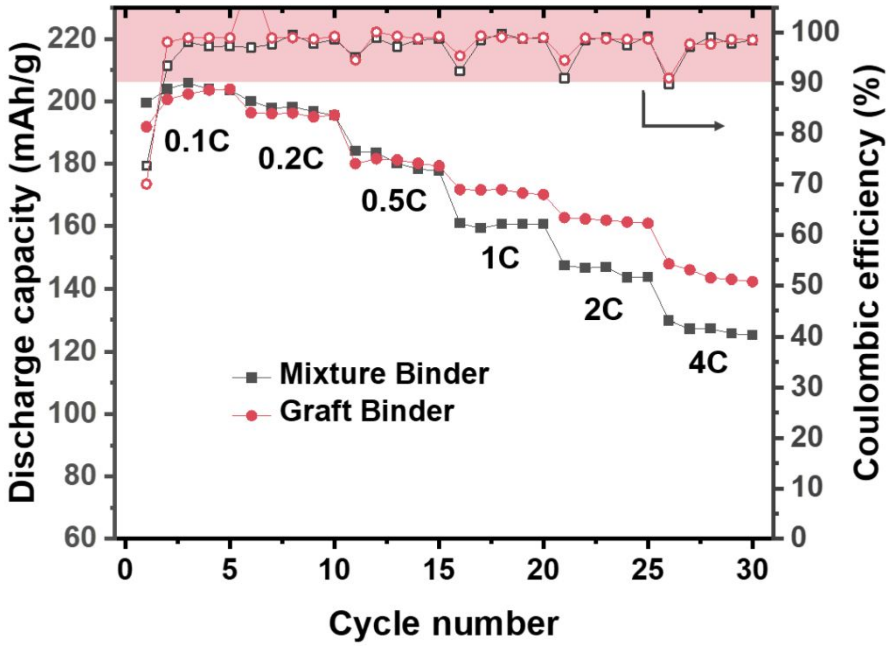

Figure S11. Rate performance of Li|NCM811 cells with composition of NCM811/carbon black/binder = 93/4/3 (wt%) using physical blend of PVDF-CTFE/PEGA/PAA binder (dark grey) and covalently grafted PVDF-CTFE-*g*-PEGA-co-PAA binder (red) in the voltage range 2.9-4.3V.

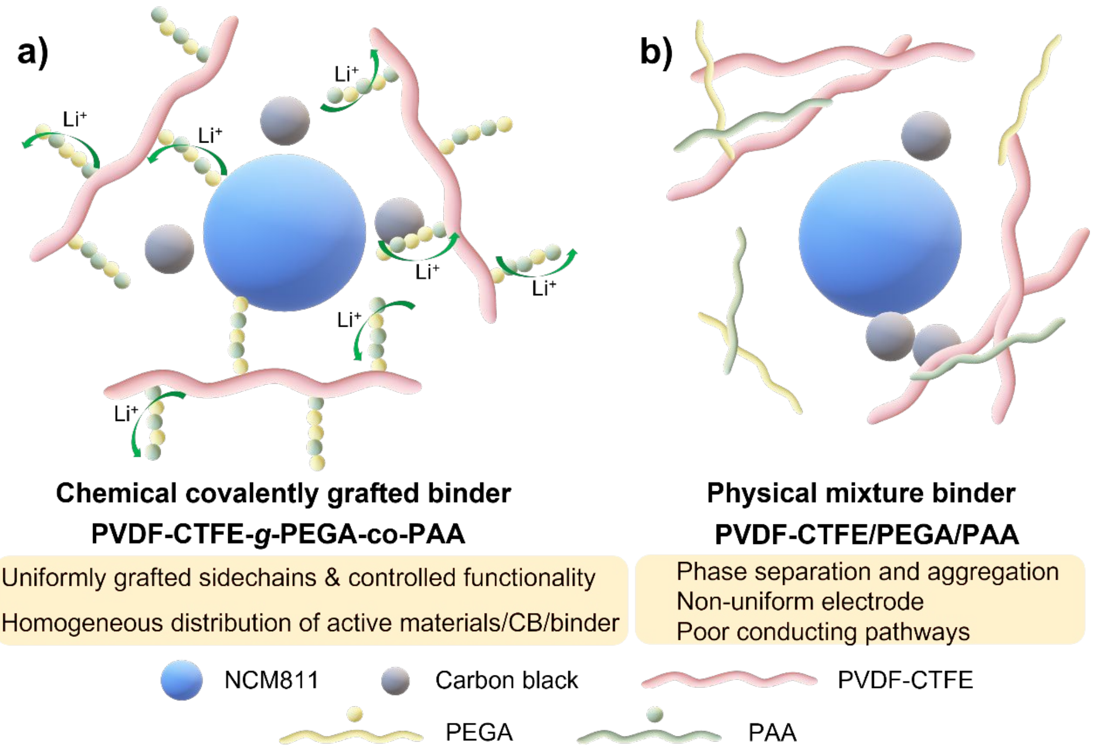

Figure S12. Scheme of NCM811 cathodes prepared with a) covalently grafted PVDF-CTFE-*g*-PEGA-co-PAA binder and b) physical mixture of PVDF-CTFE/PEGA/PAA binder using the same molecular weight and ratio of the three components.

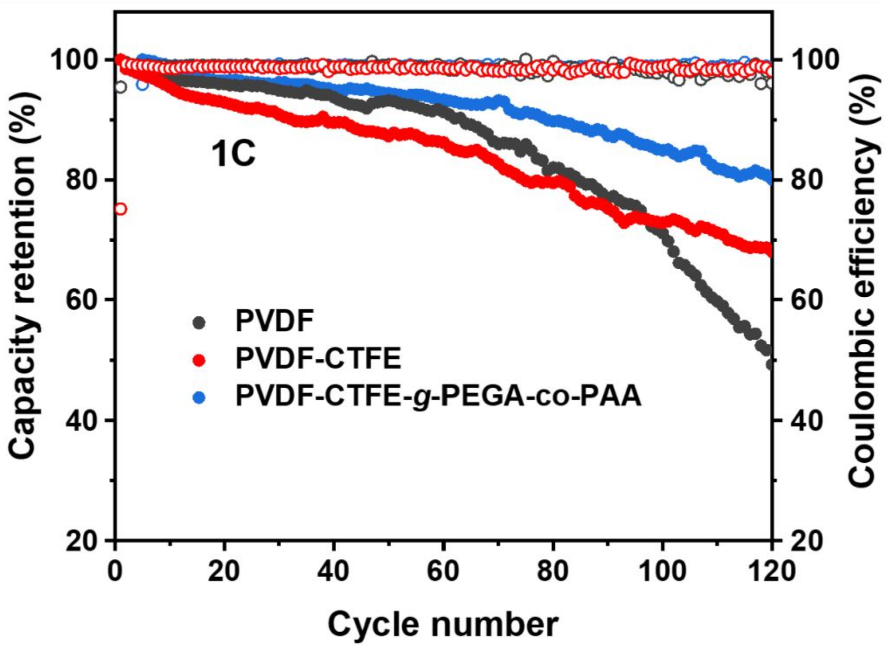

Figure S13. Capacity retention of Li|NCM811 cells using NCM811 cathodes with 3 wt% PVDF (black), PVDF-CTFE (red) and PVDF-CTFE-*g*-PEGA-co-PAA (blue) binder at 1C/1C charge/discharge. Areal capacity: ~1.1 mAh/cm^2.

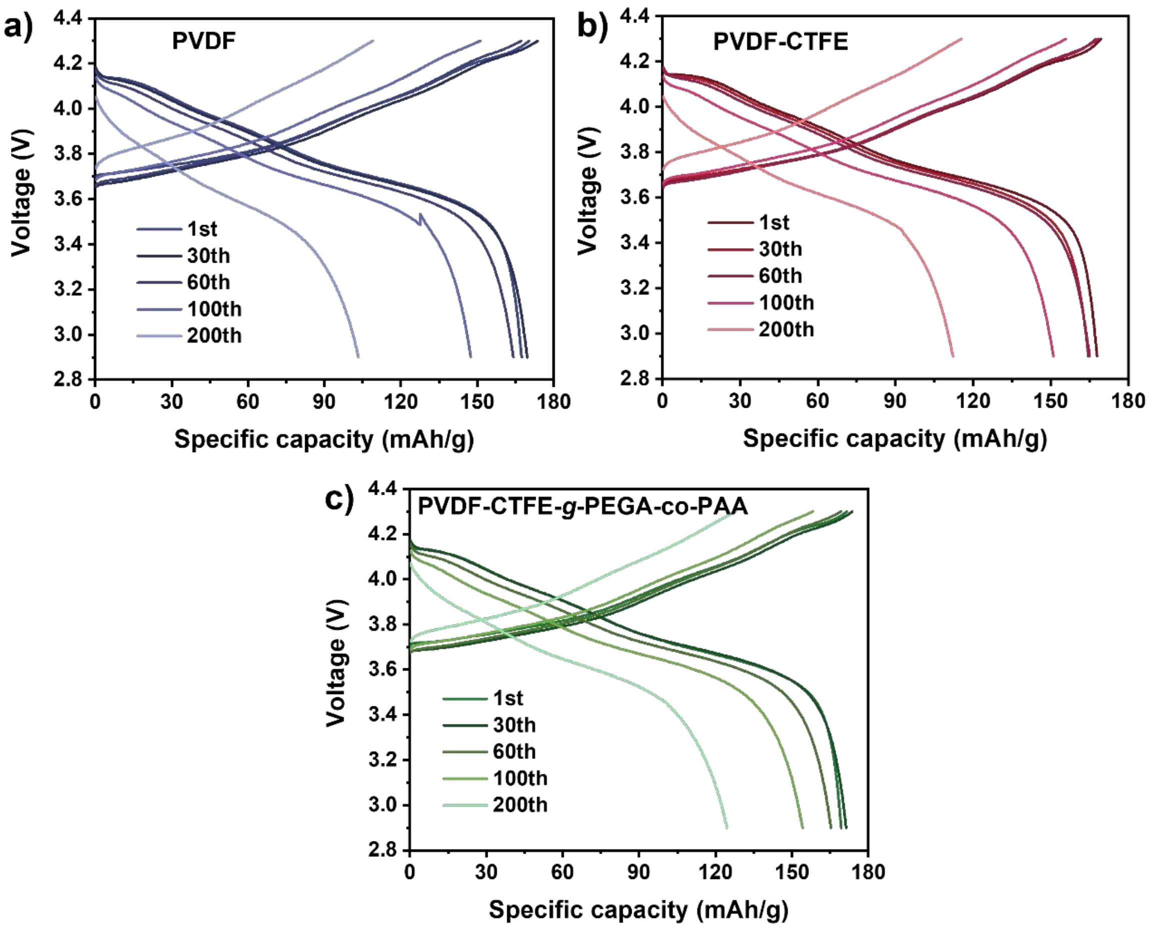

Figure S14. Voltage-capacity curves from long-term cycling of Li|NCM811 cells at 0.5C (NCM811/carbon black/binder = 93/4/3 (wt%)) with a) PVDF, b) PVDF-CTFE and c) PVDF-CTFE-*g*-PEGA-co-PAA binder.

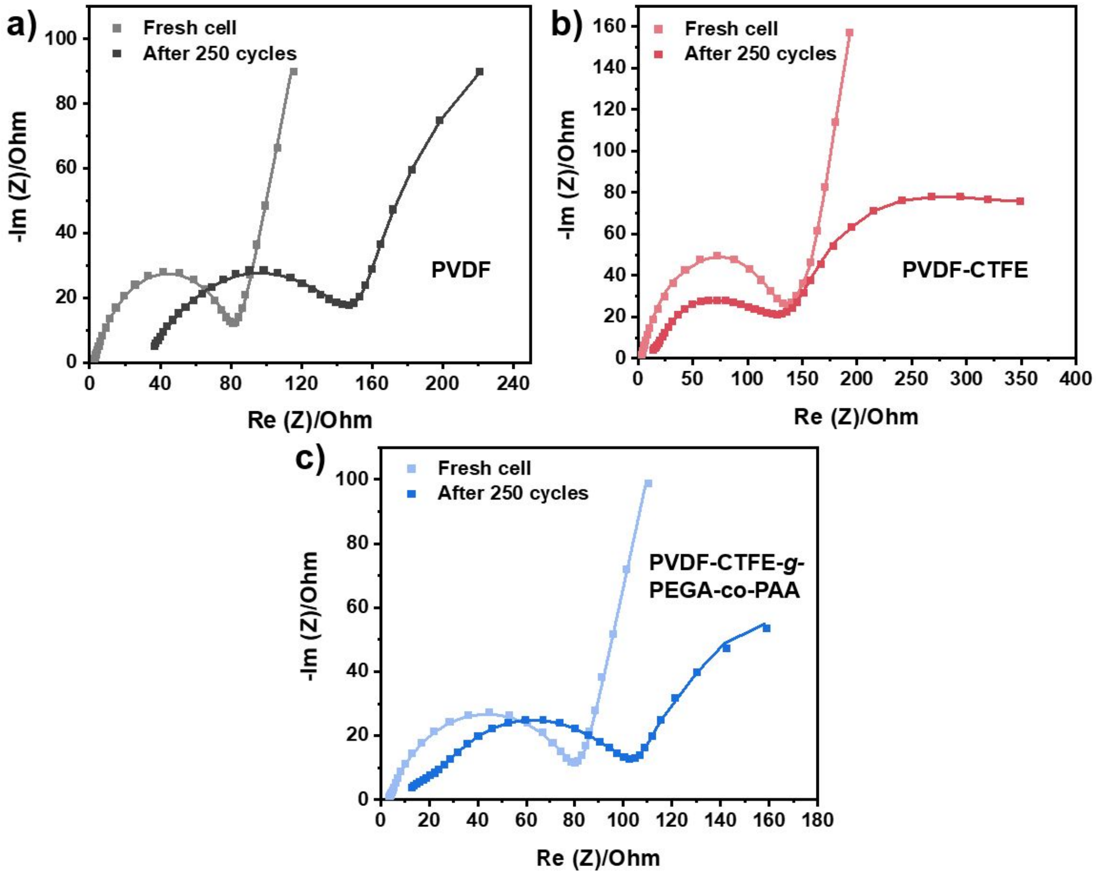

Figure S15. EIS of Li|NCM811 half cells before cycling and after 250 cycles using a) PVDF, b) PVDF-CTFE, and c) PVDF-CTFE-*g*-PEGA-co-PAA binder. Dots: original EIS data; line: fitting results.

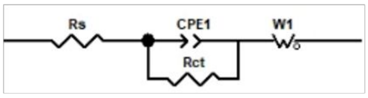

Before cycling

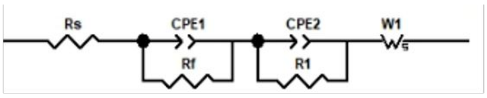

After 250 cycles

R	Before cycling			After 250 cycles		
	PVDF	PVDF-CTFE	PVDF-CTFE-*g*-PEGA-co-PAA	PVDF	PVDF-CTFE	PVDF-CTFE-*g*-PEGA-co-PAA
**Rs**	2.54 Ω	3.14 Ω	3.52 Ω	31.89 Ω	7.17 Ω	10.57 Ω
**Rf**	n/a	n/a	n/a	126.9 Ω	34.31 Ω	110.0 Ω
**Rct**	77.05 Ω	113.5 Ω	74.35 Ω	216.7 Ω	262.0 Ω	99.56 Ω

Figure S16. The equivalent circuits and the fitting results of the EIS in Figure S15.

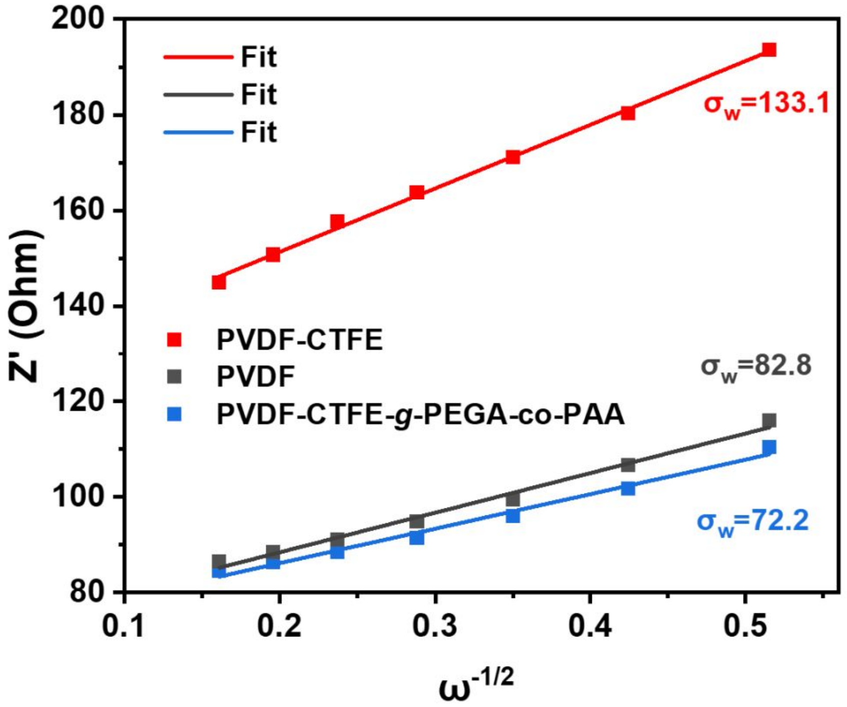

Figure S17. The Z' vs. $\omega^{-1/2}$ plots of the NCM811 cathodes with PVDF (black), PVDF-CTFE (red), PVDF-CTFE-*g*-PEGA-co-PAA (blue) binder before cycling in the low frequency region.

The D_{app} can be calculated using the following equation:

$$D_{app} = \frac{R^2T^2}{2A^2n^4F^4C^2{\sigma_w}^2}$$

n is the charge transfer number, *F* is the Faraday constant (96485 C mol^{-1}), *C* is the concentration, *A* is the electrode surface area (cm^2), *R* is the gas constant (8.314 J mol^{-1} K^{-1}), *T* is the temperature (298 K), D_{app} is the apparent diffusion coefficient (cm^2 s^{-1}), and σ_w is the Warburg coefficient (ohm·$s^{-1/2}$).

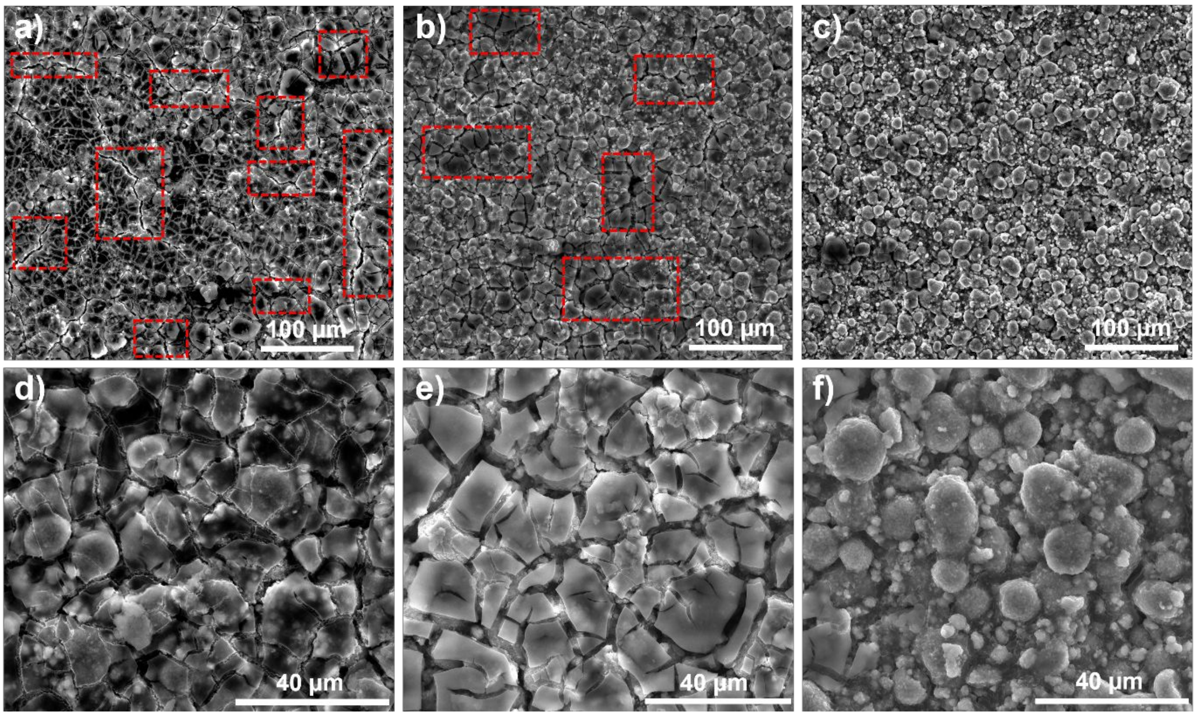

Figure S18. Top-view SEM images of NCM811 cathodes after 250 cycles with a) PVDF, b) PVDF-CTFE, and c) PVDF-CTFE-*g*-PEGA-co-PAA binder. Enlarged SEM images of NCM811 cathodes after 250 cycles with d) PVDF, e) PVDF-CTFE, and f) PVDF-CTFE-*g*-PEGA-co-PAA binder.

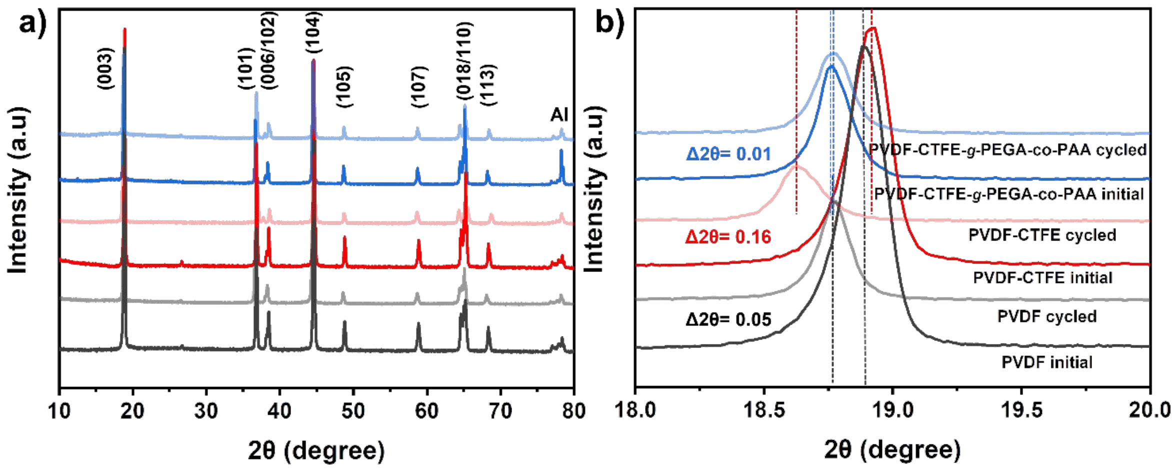

Figure S19. a) XRD patterns of NCM811 cathodes of initial and after 250 cycles in the voltage range of 2.9-4.3V with PVDF (dark grey), PVDF-CTFE (red) and PVDF-CTFE-*g*-PEGA-co-PAA (blue) binder; b) magnified XRD pattern of diffraction peak (003).

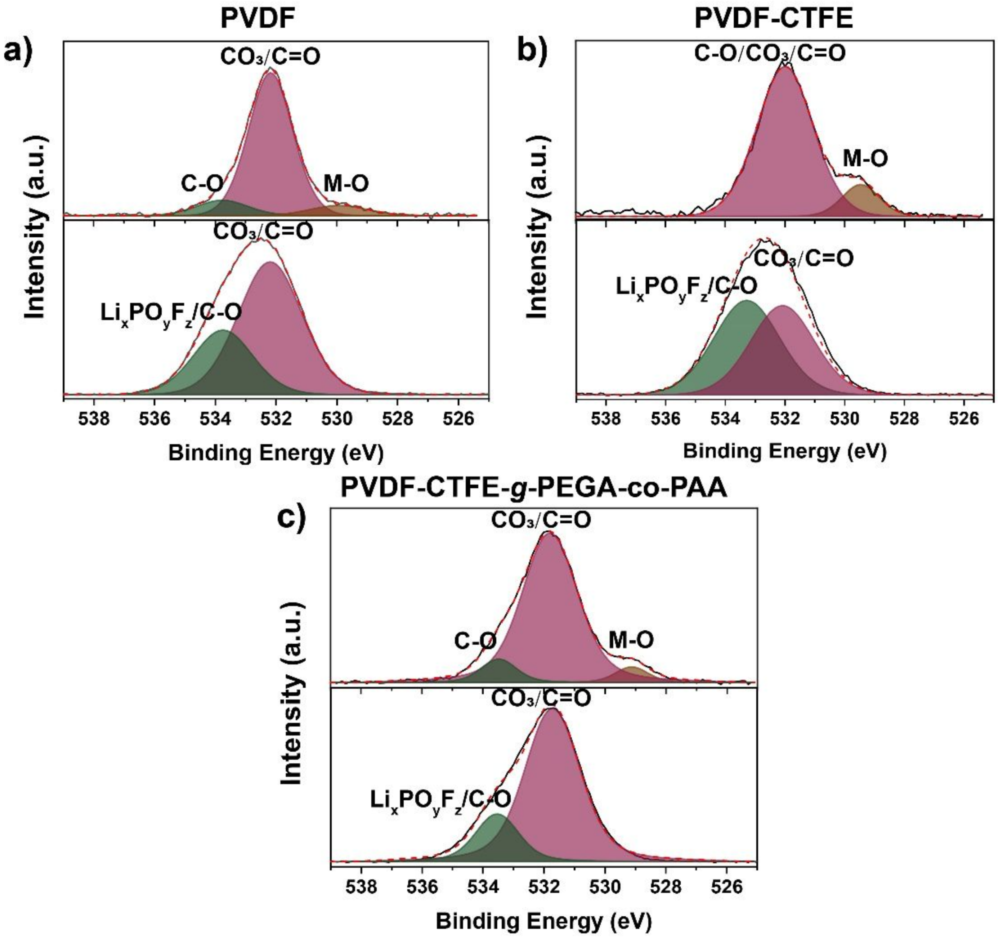

Figure S20. XPS analysis of O1s spectra of NCM811 electrodes with a) PVDF, b) PVDF-CTFE and c) PVDF-CTFE-g-PEGA-co-PAA binder before cycling (upper) and after 250 cycles (lower).
